# Actions of Pharmaceutical Lactones and Lactams on the Enzymes with Lactonase Activity: In Silico Prediction and In Vitro Study

**DOI:** 10.3390/ijms27209061

**Published:** 2026-10-13

**Authors:** Elena Efremenko, Maksim Domnin, Anastasia Sarapina, Aigul Zagidulina

**Affiliations:** Faculty of Chemistry, Lomonosov Moscow State University, Lenin Hills 1/3, Moscow 119991, Russia

**Keywords:** molecular docking, metallo-β-lactamases, lactonase, β-lactam antibiotics, statins, macrolides, warfarin

## Abstract

Pharmaceutical pollutants that contain lactone or lactam rings in their structure are frequently detected in wastewater. Enzymes with lactonase activity, synthesized by the microorganisms in aerobic activated sludge, are expected to be able to participate in the hydrolysis of these pharmaceutical pollutants, but the actual activity of many lactonases towards such compounds remains poorly characterized. In this work, a systematic molecular docking study of 12 enzymes and 39 pharmaceutical pollutants (β-lactam antibiotics, macrolides, coumarins, statins) was undertaken. The binding affinity, active-site occupancy, total enzyme surface occupancy and geometric characteristics of the molecules (the distance from the catalytic nucleophile to the carbonyl carbon of the lactone ring, and the attack angle formed between the nucleophilic atom of the enzyme and the C–N/C–O bond of the substrate) were evaluated in silico for a set of hydrolases displaying lactonase activity (AidC, AaL, hexahistidine-tagged organophosphate hydrolase (His_6_-OPH), etc.) and metallo-β-lactamases (MβLs) (NDM-1, MIM-1, SAM-1). Lactamases exhibited favorable intermolecular interaction geometry for the majority of β-lactams. No enzyme was found that satisfied the geometric criteria corresponding to the biodegradation of macrolides. With coumarins, only Paraoxonase PON2 provided an optimal geometry of biocatalysis; however, this enzyme is absent from activated sludge but may be of interest for in vivo applications. For several statins widely used in practice, combinations with enzymes having virtually ideal geometry for biocatalysis were identified: AidC/rosuvastatin lactone, AaL/lovastatin, His_6_-OPH/mevastatin, and PON2/simvastatin. For the first time, several pharmaceutical lactones were identified in silico and confirmed in vitro as inhibitors of MβL and His_6_-OPH. Competitive inhibition of NDM-1 by lactone-containing signaling molecules of Gram-negative bacteria present in activated sludge was revealed for the first time both in silico and in vitro. The obtained results‘ may be taken into account when implementing monitoring of biological stages at water treatment stations and improve the efficiency of water purification. The revealed characteristics of MβL inhibition by macrolides may be taken into account as one of possible explanations for the effective application of β-lactams in combinations with macrolides known from multiple paratactical investigations.

## 1. Introduction

**Figure 1:**
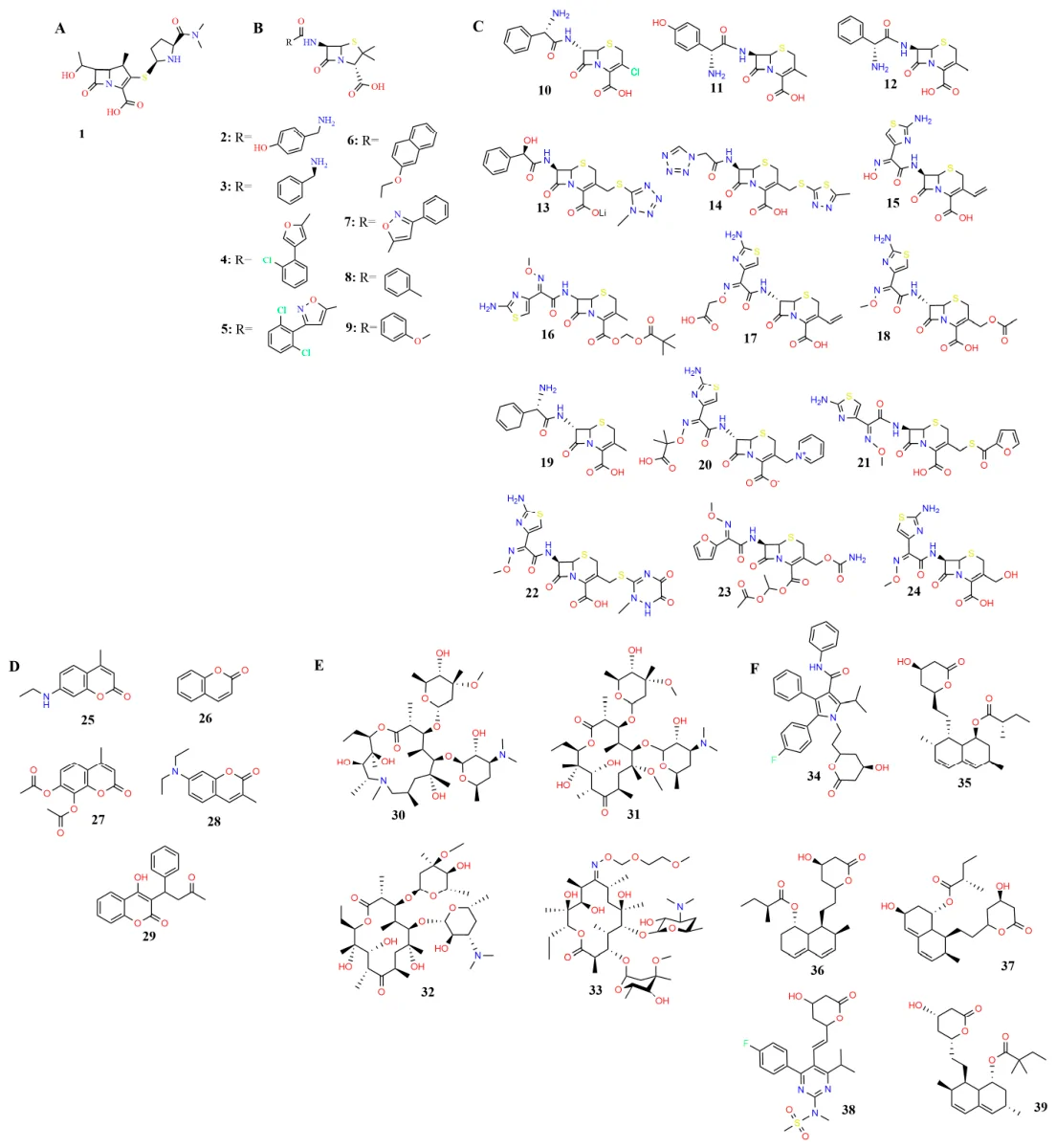
Molecules of lactone-containing pharmaceutical pollutants detected in wastewater: (**A**) carbapenems (1—meropenem); (**B**) penicillins (2—amoxicillin, 3—ampicillin, 4—cloxacillin, 5—dicloxacillin, 6—nafcillin, 7—oxacillin, 8—penicillin G, 9—penicillin V); (**C**) cephalosporins (10—cefaclor, 11—cefadroxil, 12—cefalexin, 13—cefamandole lithium, 14—cefazolin, 15—cefdinir, 16—cefetamet pivoxyl, 17—cefixime, 18—cefotaxime, 19—cefradine, 20—ceftazidime, 21—ceftiofur, 22—ceftriaxone, 23—cefuroxime axetil, 24—desacetylcefotaxime); (**D**) coumarins (25—C47T1 (4-methyl-7-ethylaminocoumarin), 26—coumarin, 27—DAMC (7,8-diacetoxy-4-methylcoumarin), 28—DEAMC (7-(diethylamino)-4-methylcoumarin), 29—warfarin); (**E**) macrolides (30—azithromycin, 31—clarithromycin, 32—erythromycin, 33—roxithromycin); (**F**) statins (34—atorvastatin, 35—lovastatin, 36—mevastatin, 37—pravastatin, 38—rosuvastatin lactone, 39—simvastatin).

The current intensive development of the bioeconomy is accompanied by a number of concomitant environmental problems, one of which is the release of various pharmaceutical pollutants into environment [1]. These compounds can exert negative effects on members of natural ecosystems, as well as on the microbial consortia employed for biological water treatment at wastewater treatment plants [2]. Among the most frequently detected pharmaceutical contaminants are β-lactam antibiotics, including carbapenems, penicillins and cephalosporins, all of which contain a β-lactam ring (Figure 1).

They belong to the class of antimicrobial agents most widely used in clinical practice owing to their fairly broad spectrum of activity and specificity for bacterial cells. Macrolide antibiotics, which contain a macrocyclic lactone ring, together with statins, widely applied as cholesterol-lowering drugs, and coumarins, which are unsaturated aromatic lactones used as medicinal agents, are likewise among the pharmaceutical pollutants frequently detected worldwide [3,4,5]. All of these compounds share a common structural feature, namely a lactam or lactone ring.

It is known that for many bacterial cells, and even fungi participating in wastewater treatment, lactone-containing compounds serve as autoinducers of their Quorum Sensing (QS) systems, which are manifested through the activation of processes responsible for the development of cellular resistance to various adverse factors, including antibiotic exposure [6].

**Figure 2:**
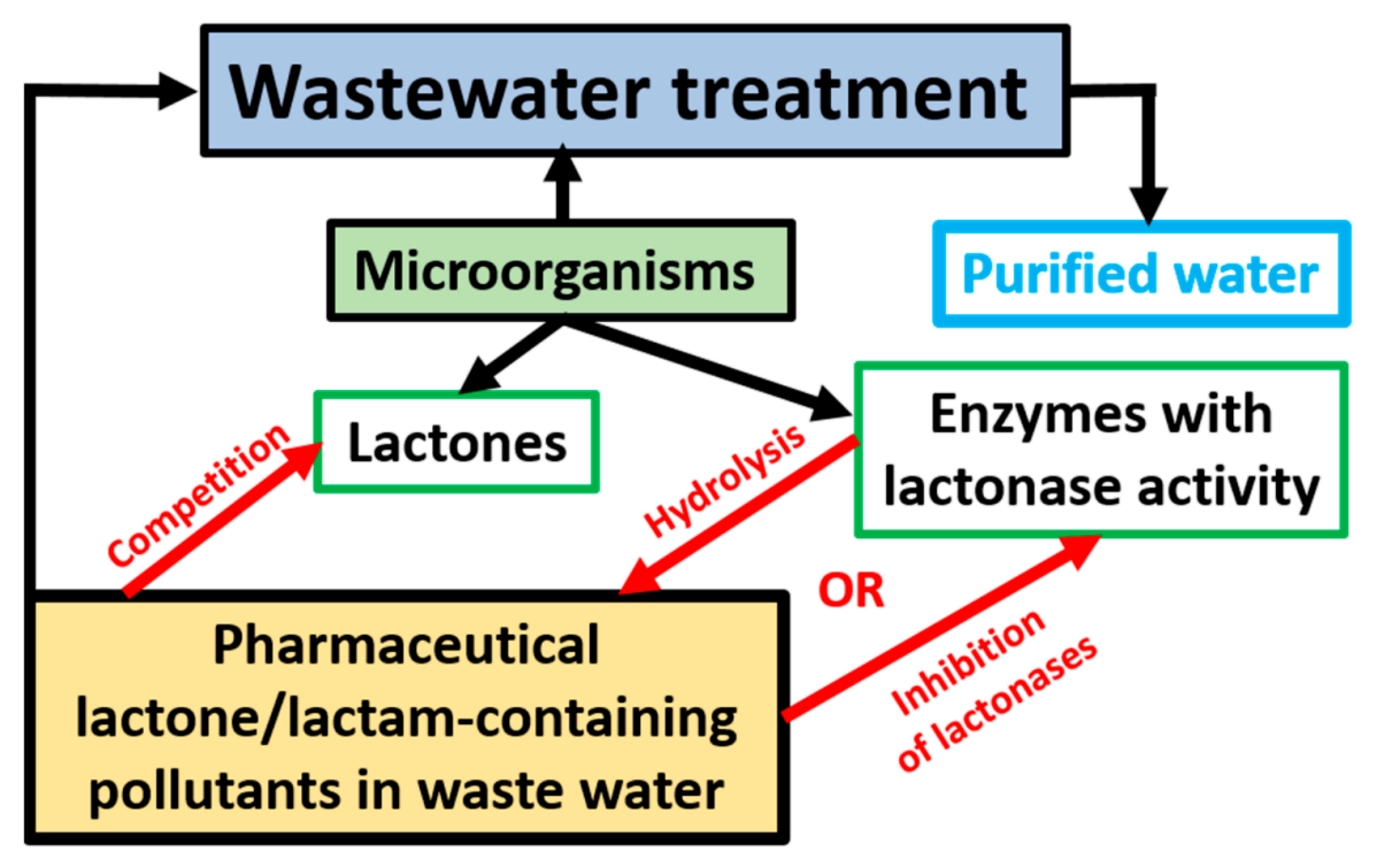
Schematic representation of how pharmaceutical pollutants can affect microorganisms and their enzymes sensitive to lactone/lactam-containing pharmaceutical compounds.

The presence of such pharmaceutical pollutants in the microenvironment (Figure 2), which can theoretically act as analogues of QS autoinducers, may lead to a reduction in the functional activity and stability of the microbial populations involved in water purification [7,8,9]. Biological stages of treatment of wastewater containing such pharmaceutical pollutants may result in their accumulation inside microbial cells, with a subsequent decline in the productivity and efficiency of the biological treatment process.

However, bacteria and fungi participating in wastewater treatment frequently possess enzymes with lactonase activity (Table 1 [9,10,11,12,13,14,15,16,17,18,19]), whose action is directed towards the hydrolysis of lactone-containing autoinducer molecules of competing microbial cells (Figure 2). It has been demonstrated that β-lactams are capable of binding to lactonases at the active site: in particular, molecular docking revealed the binding of penicillin G to AHL-lactonase AiiA [20], while certain metallo-β-lactamases (MβLs) are able to hydrolyze lactones, which may be indicative of enzymatic promiscuity (non-specific activity) within the MβL fold family [13].

The use of these enzymes for the degradation of lactone/lactam-containing pharmaceutical pollutants appears feasible; however, this requires an investigation of the possible interactions between these molecules (Figure 1) and various known lactonases (Table 1 [9,10,11,12,13,14,15,16,17,18,19]).

Enzymatic hydrolysis of lactones and lactams is known to result in ring opening and the consequent loss of biological activity and toxicity of the respective pharmaceutical compounds [21]. Enzymes belonging to the metallo-β-lactamase (MβL) superfamily, which are characterized by conserved metal-binding motifs and display broad functional diversity, are of particular interest. Beyond their canonical lactamase activity, these enzymes can function as lactonases in reactions with some lactones [22]; however, it has never been confirmed that MβLs can hydrolyze lactone-containing pharmaceuticals or N-acyl homoserine lactones (AHLs)—molecules involved in bacterial Quorum Sensing of Gram-negative bacteria [15].

Lactonases are enzymes capable of hydrolyzing various lactones, and they have therefore attracted research attention for tackling the degradation of lactone-containing compounds [14]. However, the substrate specificity of known lactonases towards structurally diverse pharmaceutical pollutants, including macrolides, coumarins, and statins, which may act not only as potential substrates but also as inhibitors of these enzymes, remains poorly investigated. This information is important, because the biosynthesis of the enzymes can be purposefully stimulated within biological wastewater treatment if the enzymes possess hydrolytic activity towards the mentioned lactone-containing pollutants. Otherwise, a decrease in the efficiency of wastewater treatment at the biological stage can be observed if the discussed compounds interact with the known lactonases as inhibitors.

The present study was aimed at addressing this knowledge gap through a systematic assessment of the ability of different pharmaceutical compounds containing lactone and lactam rings to interact with hydrolytic enzymes possessing lactonase activity towards QS molecules from various microorganisms. The study employed the pharmaceutical pollutants (Figure 1) and enzymes (Table 1) listed above as the objects of study.

In the present work, 12 enzymes (Table 1) were employed, including different MβLs (NDM-1, MIM-1, SAM-1), lactonases (AaL, AidC, AiiA, AiiB, SsoPox, ZEN-lactonase), and other hydrolases with lactonase activity (PON2, His_6_-OPH, OTase), which are hypothetically capable of binding 39 pharmaceutical pollutants containing lactone and lactam rings (Figure 1). Among them were 24 β-lactam antibiotics (8 penicillins, 15 cephalosporins and the carbapenem meropenem), 4 macrolide antibiotics (azithromycin, clarithromycin, erythromycin, roxithromycin), 5 coumarins (coumarin, DAMC, DEAMC, warfarin, and C47T1), and 6 statins (atorvastatin, lovastatin, mevastatin, pravastatin, rosuvastatin lactone, simvastatin).

It is well confirmed that computational modeling of intermolecular contacts allows evaluation not only of the interaction energy but also of the geometric parameters of binding, as well as identification of the involvement of enzyme catalytic sites in these processes, which is critically important prior to experimental validation. We have previously demonstrated the successful application of in silico molecular docking to study enzyme–substrate interactions and to predict possible substrate spectra of lactonases or their inhibitors [14].

The parameters assessed by molecular docking, namely, occupation of active-site area by the ligands (pharmaceutical pollutants), the total enzyme surface area occupied by them, their interaction energy with enzyme active sites, the distance between the nucleophilic atom (the oxygen of the catalytic serine or of a metal-coordinated water molecule) and the electrophilic carbonyl carbon atom of the lactone/lactam ring, as well as the nucleophilic attack angle, which defines the trajectory of the ligand approaching the reaction center [23], are critically important for the prediction of biocatalysis. These parameters were assessed in this work by bioinformatic analysis with further biocatalysis checking in vitro.

Visualization of the results of the intermolecular interaction study for all enzyme–ligand combinations, presented as a heatmap, allowed the identification of distinct patterns of potential substrate specificity, as well as enzyme–ligand pairs in which inhibition of the enzymes by the investigated pharmaceutical lactone-containing compounds is possible. Determination of in vitro inhibition constants revealed the influence of lactone-containing pharmaceutical compounds with different therapeutic applications on enzymes that represent classical lactamases and lactonases.

## 2. Results

To assess the potential hydrolytic activity of 12 enzymes towards 39 pharmaceutical compounds by molecular docking, not only standard binding parameters (affinity, surface areas of intermolecular interactions) were analyzed, but also the geometric characteristics that determine the catalytic competence of the complex.

The key criterion for evaluating the feasibility of enzymatic hydrolysis of the selected compounds was whether the geometry of their binding in the enzyme active site corresponded to parameters favoring nucleophilic attack on the carbonyl carbon atom of the lactone/lactam ring. Within the framework of the performed molecular modeling, two fundamental parameters were calculated for each enzyme–ligand complex: the distance from the nucleophilic oxygen atom of the catalytic serine or the metal atom coordinating a water molecule as a nucleophile to the electrophilic carbonyl carbon of the lactone/lactam ring, as well as the nucleophilic attack angle, which defines the trajectory of approach to the reaction center.

The values of these parameters for the best poses from the docking results were divided into several separate groups and analyzed according to the specific structure of the pharmaceutical pollutants.

### 2.1. Evaluation of the Interactions of β-Lactam Pollutants with Enzymes

**Figure 3:**
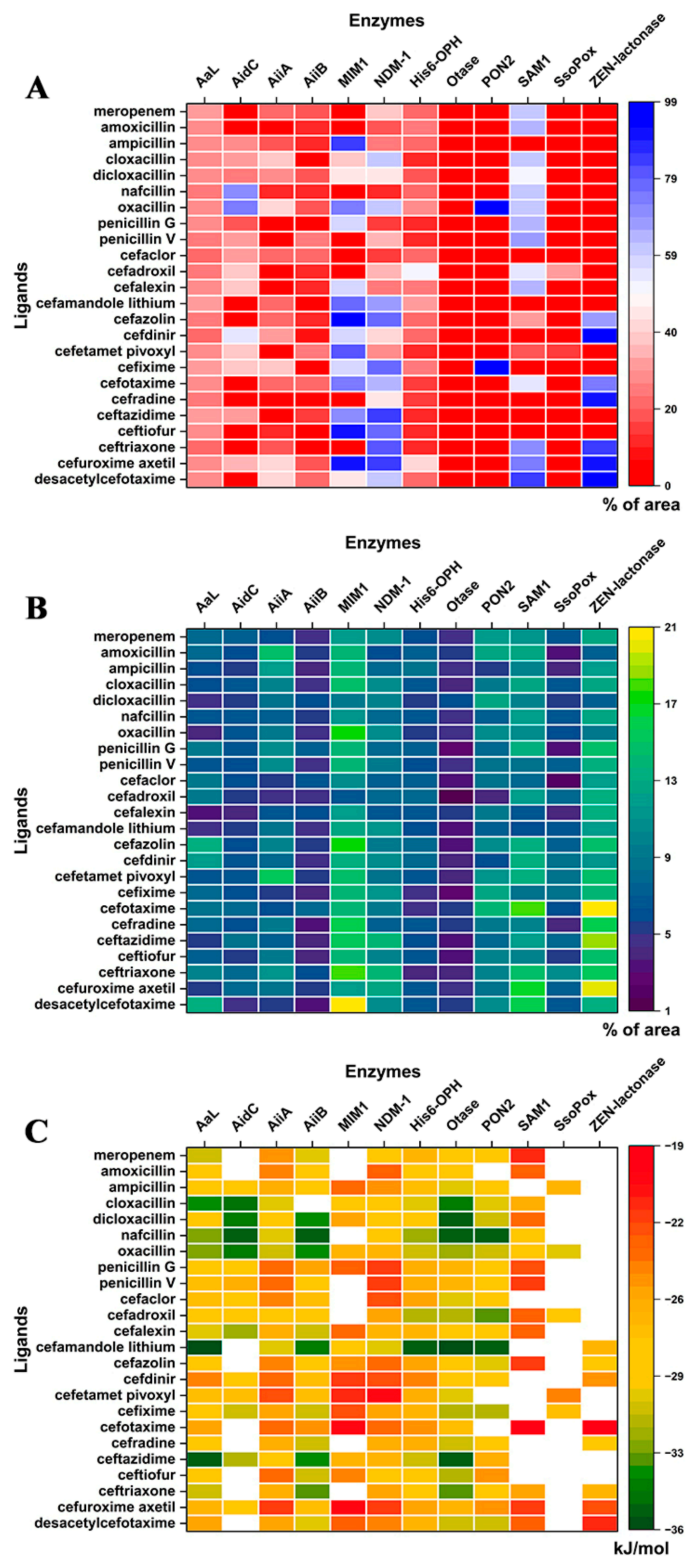
Surface area (%) occupied by β-lactam antibiotic molecules on the enzyme surface in the region of their active sites (**A**) or on the entire enzyme surface (**B**). The surface area of the enzyme active sites themselves (**A**) or the entire enzyme surface (**B**) was taken as 100%. Also shown are the binding affinity (kJ/mol) of β-lactam antibiotic molecules with the enzyme surface in the active site region (**C**).

Interaction models of 12 enzymes with 24 β-lactam antibiotics (the carbapenem meropenem, 8 penicillins, and 15 cephalosporins) were obtained (Figure S1–S12). The results of simulated interactions are presented in Figure 3.

The highest active-site occupancy was observed for the MβLs MIM-1, NDM-1, an SAM-1, as well as for ZEN-lactonase. For MIM-1 with cefazolin, it reached 93%, and with cefuroxime axetil, 92%; for ZEN-lactonase with cefradine, 92%, with cefazolin, 66%, and with cefotaxime, 73%. For SAM-1, penicillins (amoxicillin, ampicillin, penicillin G) occupied 64 to 68% of the active site, whereas for the other MβLs, cephalosporins displayed a higher probability of being localized in the catalytic center region. For the majority of ligands with the listed enzymes, the binding affinities were less than –28 kJ/mol (strong binding). The highest affinity was observed for OTase with dicloxacillin (–35.1 kJ/mol) and cefamandole (–36.0 kJ/mol), and for AidC with oxacillin (–34.2 kJ/mol), which corresponds to strong binding according to the criteria [24,25].

Analysis of the molecular docking results of β-lactam antibiotics into the active sites of enzymes of the MβL family (NDM-1, MIM-1, SAM-1) showed that a significant proportion of the ligands are localized in the active-site region, exhibiting geometric parameters close to those optimal for a hydrolysis reaction to proceed. In particular, the distances from the carbonyl carbon atom of the lactam ring to the metal ions coordinating an activated water molecule, as well as the corresponding nucleophilic attack angles, satisfy the criteria for productive binding.

**Figure 4:**
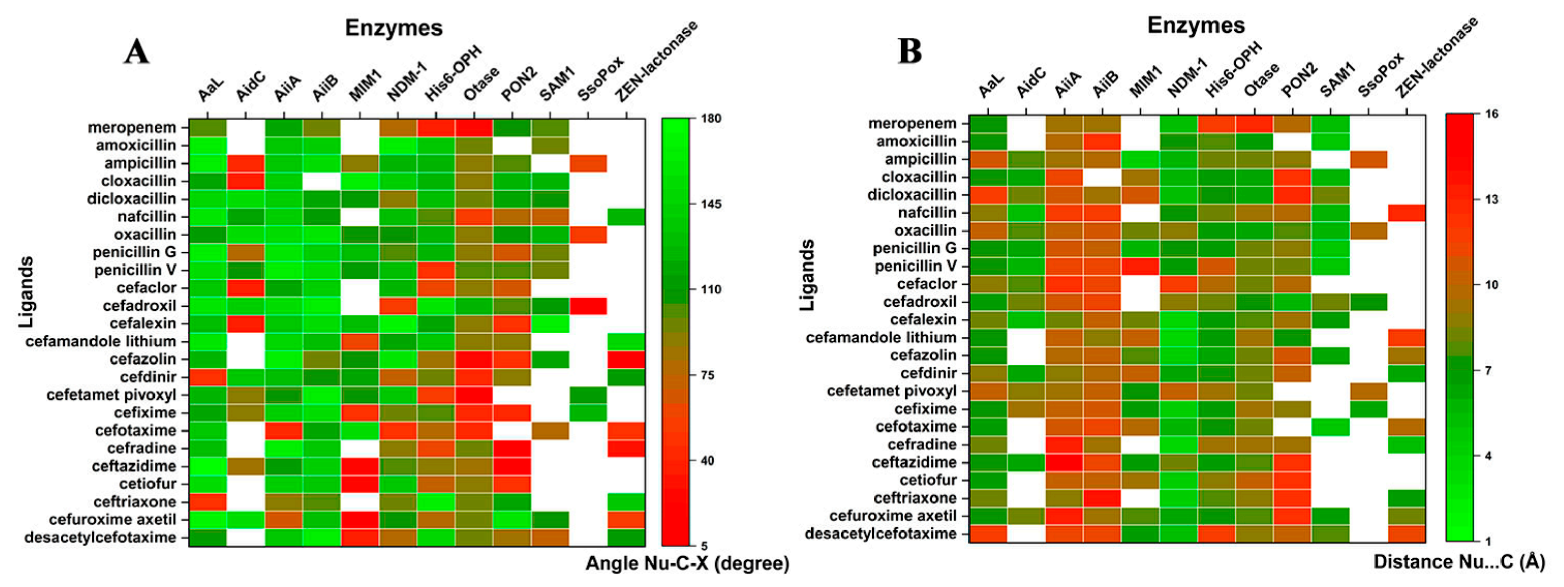
Calculated nucleophilic attack parameters for enzyme/β-lactam complexes. Shown are (**A**) attack angles (degrees) defining the trajectory of substrate approach to the reaction center (Nu–C–X, where X = O, N for lactone and lactam, respectively) and (**B**) distances from the nucleophile to the electrophilic carbonyl carbon of the lactone/lactam ring (it is marked as Nu…C). The most favorable values of the geometric parameters for the catalytic reaction to proceed are indicated in green, and the least favorable are shown in red. Cases in which the ligands are distant from the enzyme active site are uncolored.

Lactonases of the MβL superfamily (AaL, AidC, AiiA, AiiB) generally did not provide angles close to 180° (Figure 4). The maximum recorded values were 168° (AaL with penicillin G, distance 7.4 Å), 155.8° (AidC with dicloxacillin, distance 8.1 Å), 167.2° (AiiA with cefazolin, 9.6 Å), and 168.3° (AiiB with cefetamet pivoxil, 10.3 Å), although the distances were far from optimal. ZEN-lactonase, despite high active-site occupancy with a number of cephalosporins, exhibited attack angles in the range of 33.5–147.3°, which precludes the possibility of a linear attack. SsoPox also did not form angles > 150° with β-lactams.

### 2.2. Evaluation of the Interactions of Macrolides with Enzymes

**Figure 5:**
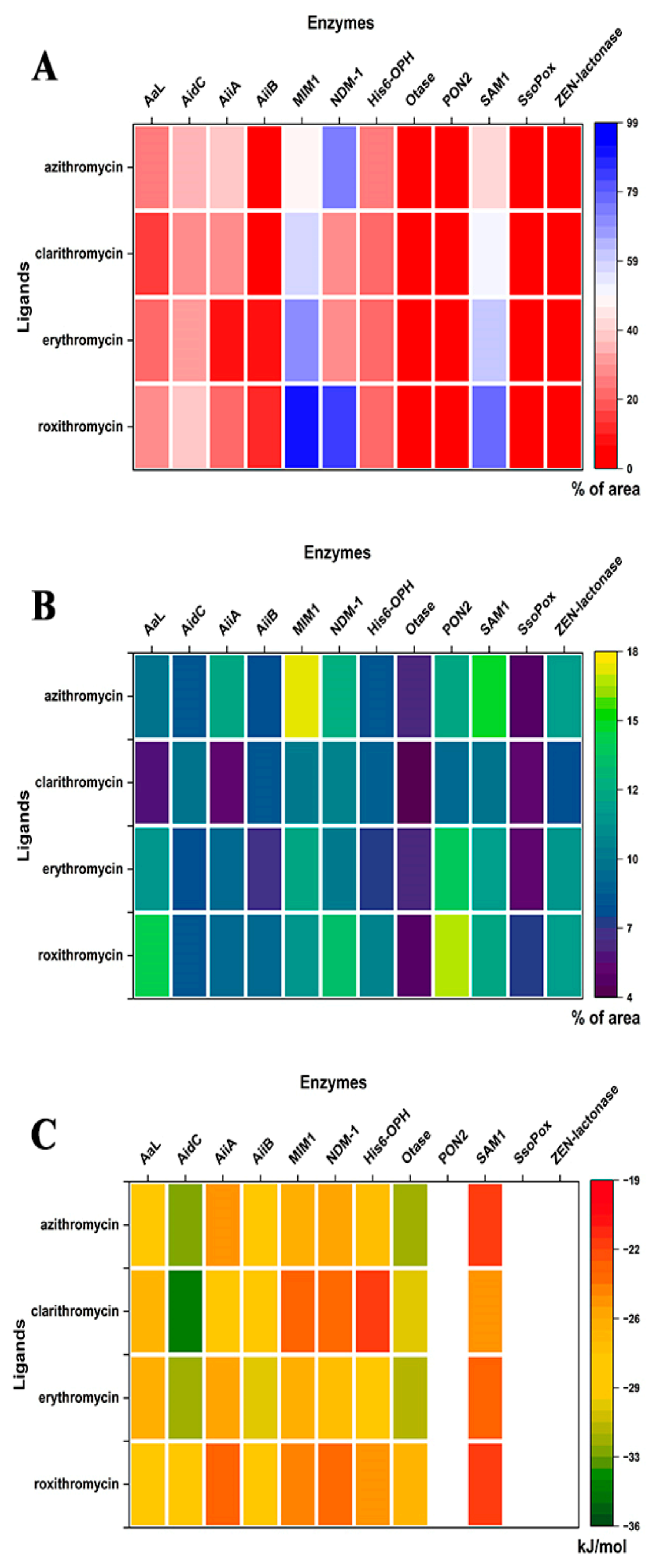
Surface area (%) occupied by macrolide antibiotic molecules on the enzyme surface in the region of their active sites (**A**) or on the entire enzyme surface (**B**). The surface area of the enzyme active sites themselves (**A**) or the entire enzyme surface (**B**) was taken as 100%. Also shown are the binding affinity (kJ/mol) of macrolide antibiotic molecules with the enzyme surface in the active site region (**C**).

**Figure 6:**
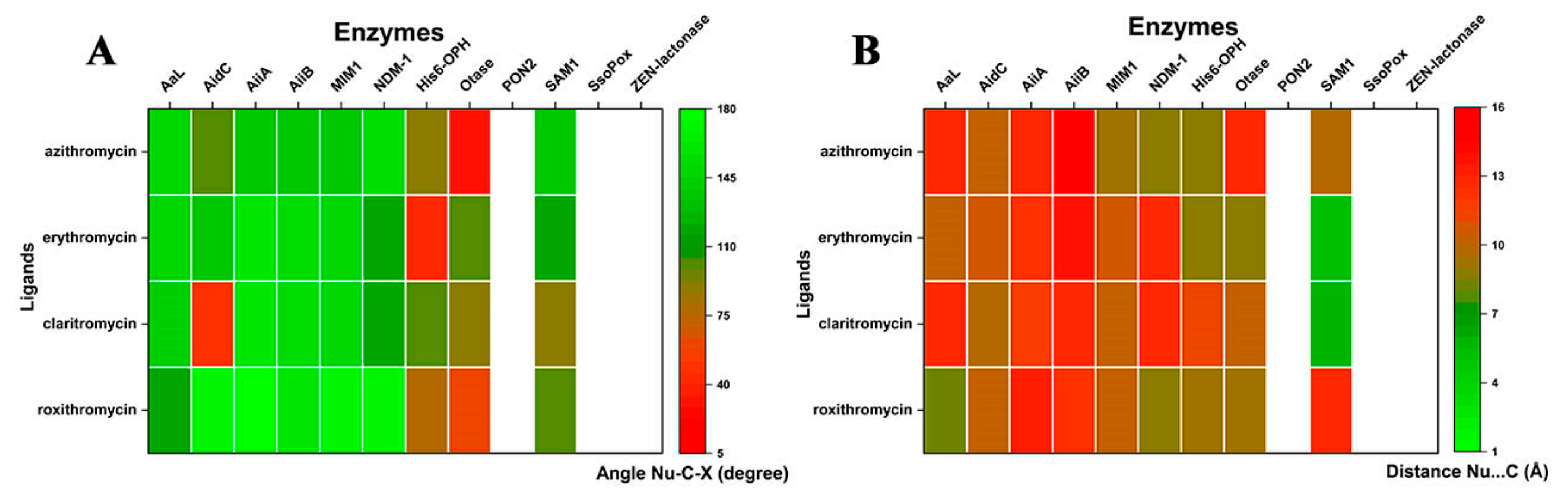
Calculated nucleophilic attack parameters for enzyme–macrolide complexes. Shown are (**A**) attack angles (degrees) defining the trajectory of substrate approach to the reaction center (Nu–C–X, where X = O, N for lactone and lactam, respectively) and (**B**) distances from the nucleophile to the electrophilic carbonyl carbon of the lactone/lactam ring (it is marked as Nu…C). The most favorable values of the geometric parameters for the catalytic reaction to proceed are indicated in green, and the least favorable are shown in red. Cases in which the ligands are distant from the enzyme active site are uncolored.

Interaction models of the same 12 enzymes with four macrolide antibiotics (azithromycin, clarithromycin, erythromycin, roxithromycin) were investigated (Figure S1–S12), and the obtained results were statistically treated and are presented in Figure 5 and Figure 6.

The highest proportion of active-site occupancy was observed for the MβLs MIM-1, NDM-1, and SAM-1 (Figure 5). In the case of roxithromycin, it reached 92, 86, and 79%, respectively; for erythromycin, with MIM-1 and SAM-1—70 and 62%.

AidC and NDM-1 also bound macrolides in the active-site region, although the occupied area was substantially lower (25–37%). AiiB, SsoPox, and ZEN-lactonase practically did not accumulate macrolides in the catalytic center region (0–25%). For OTase and PON2, not a single instance of localization in the active-site region was observed. The highest affinity was recorded for AidC in complexes with clarithromycin (–34.3 kJ/mol) and azithromycin (–32.6 kJ/mol), as well as for OTase with azithromycin (–31.7 kJ/mol). However, for OTase the ligands were not localized in the active site. The classical MβLs (MIM-1, NDM-1, SAM-1) exhibited moderate affinity (from –21.9 to –27.0 kJ/mol), while His_6_-OPH showed the lowest values (from –21.8 to –27.8 kJ/mol), which corresponds to weak binding [24,25].

Analysis of the geometric parameters critical for nucleophilic attack demonstrated that none of the complexes achieved a combination of distance and angle close to optimal for hydrolysis of the lactone ring (Figure 6).

The minimum distances between the nucleophilic oxygen and the carbonyl carbon of the lactone were 5.3 Å (SAM-1–erythromycin) and 5.7 Å (SAM-1–clarithromycin). In the vast majority of cases, the distances exceeded 8 Å. Attack angles ranged from 31.6° (OTase–azithromycin) to 174.5° (AiiA–roxithromycin). For the few combinations in which the value of the nucleophile–carbonyl carbon angle fell within a favorable range, the reaction centers did not approach to the required distance.

Thus, despite moderate affinity and, for a number of MβLs, substantial shielding of the active site, the geometric parameters rule out the possibility of efficient hydrolysis of macrolide antibiotics by any of the investigated enzymes.

### 2.3. Evaluation of Interactions of Coumarin Pharmaceutical Pollutants with Enzymes

**Figure 7:**
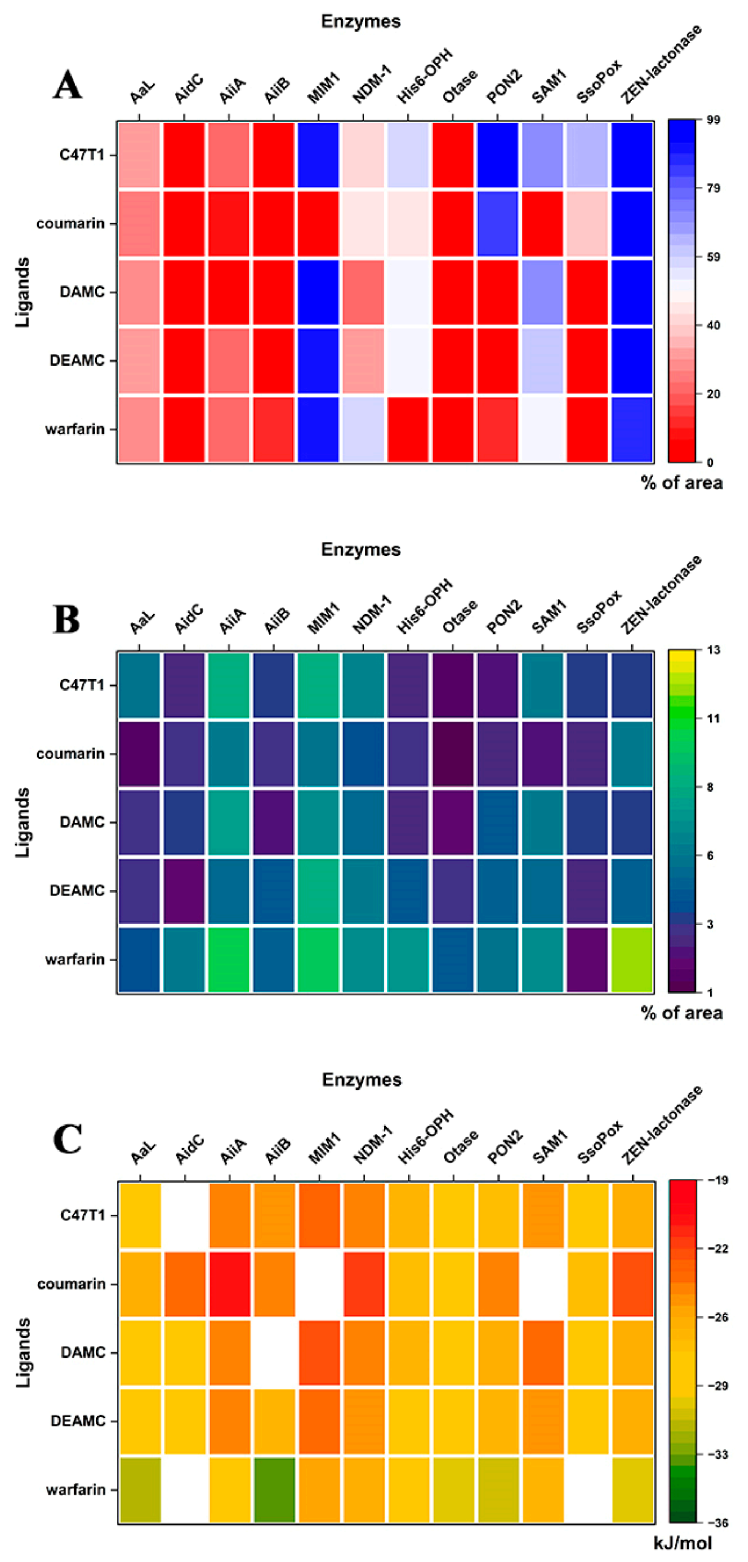
Surface area (%) occupied by coumarin molecules on the enzyme surface in the region of their active sites (**A**) or on the entire enzyme surface (**B**). The surface area of the enzyme active sites themselves (**A**) or the entire enzyme surface (**B**) was taken as 100%. Also shown are the binding affinity (kJ/mol) of coumarin molecules with the enzyme surface in the active site region (**C**).

**Figure 8:**
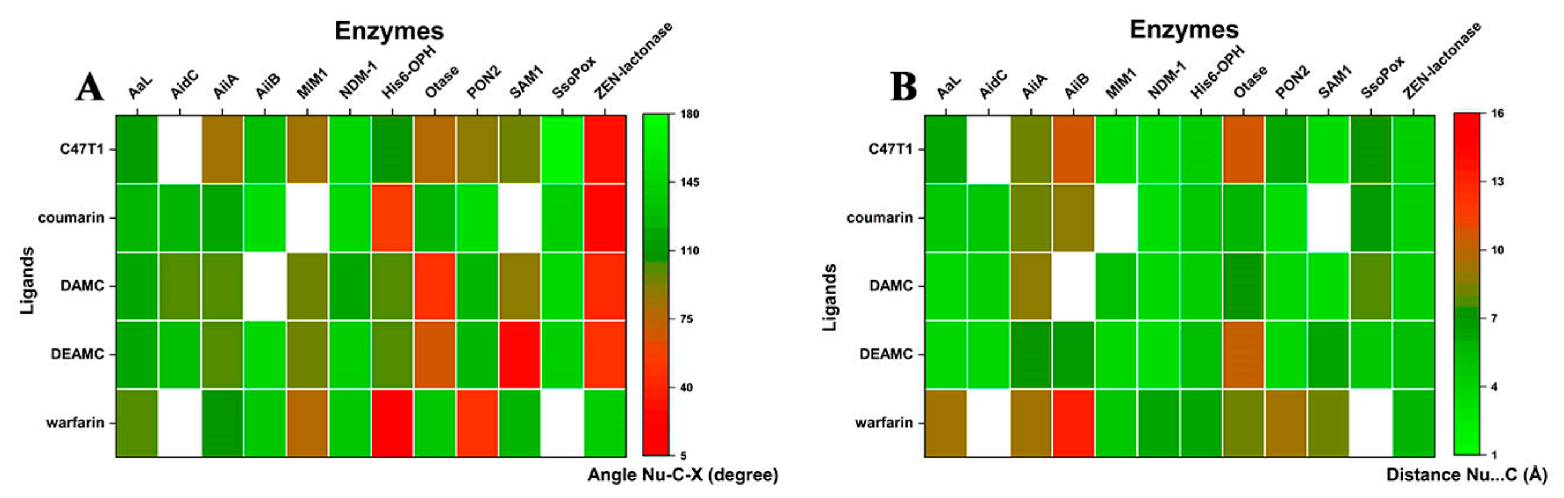
Calculated nucleophilic attack parameters for enzyme–coumarin complexes. Shown are (**A**) attack angles (degrees) defining the trajectory of substrate approach to the reaction center (Nu–C–X, where X = O, N for lactone and lactam, respectively), and (**B**) distances from the nucleophile to the electrophilic carbonyl carbon of the lactone/lactam ring (it is marked as Nu…C). The most favorable values of the geometric parameters for the catalytic reaction to proceed are indicated in green, and the least favorable are shown in red. Cases in which the ligands are distant from the enzyme active site are uncolored.

Interaction models of the 12 investigated enzymes with five coumarins (coumarin, DAMC, DEAMC, C47T1, warfarin) were obtained in silico (Figure S1–S12). The results of these interaction simulations are summarized in Figure 7 and Figure 8.

These ligands occupied a significant proportion of the active-site area in the MβLs (MIM-1, NDM-1, SAM-1), as well as in ZEN-lactonase and His_6_-OPH (Figure 7). For C47T1 and DAMC with MIM-1, this value reached 92–93%; for ZEN-lactonase with DAMC and DEAMC, it reached 98.8%, and for coumarin, 95.2%. In His_6_-OPH, coumarins occupied from 45.6% (coumarin) to 58.9% (C47T1) of the active-site area. AaL and PON2 also accumulated small coumarins in the catalytic pocket: for AaL the occupancy was 24–30%, while for PON2 coumarin occupied 85.4%. In contrast, AidC, OTase, and SsoPox practically did not bind coumarins in the active-site region (0–2.9%).

The highest affinity was observed for OTase with warfarin (–29.9 kJ/mol) and DEAMC (–29.6 kJ/mol), for SsoPox with C47T1 (–29.5 kJ/mol) and DEAMC (–28.9 kJ/mol), as well as for AaL with warfarin (–31.2 kJ/mol) and C47T1 (–28.2 kJ/mol). All these values correspond to strong binding according to the applied criteria [24,25]. However, high affinity did not always coincide with favorable geometry for the catalytic act to proceed. In particular, OTase and SsoPox, while exhibiting strong binding, had large distances from the nucleophile to the carbonyl carbon atom of the lactone ring (over 7 Å) and/or non-optimal attack angles, which suggests that productive binding for catalysis is unlikely under the modeled conditions.

Analysis of the distances and nucleophilic attack angles, taking into account the criterion that an angle approaching 180° is necessary for efficient hydrolysis, substantially narrowed the set of potentially active pairs (Figure 8). The only combination in which a short distance (3.4 Å) and an angle approaching a linear attack (152.1°) were combined was found for PON2 with coumarin. For NDM-1 in complex with coumarin, the distance was 3.0 Å at an angle of 145.5°, which can be considered a borderline case. The remaining pairs, including AaL, AidC, MIM-1, and SAM-1, which appeared promising under less stringent criteria (angles 90–130°), in reality do not satisfy the requirement for a linear attack—their angles do not exceed 130°, which precludes productive positioning of the nucleophile relative to the C–O bond of the lactone.

For C47T1, the maximum value of the nucleophile–carbonyl carbon–oxygen atom angle was 172.1° (SsoPox); however, the distance to the carbonyl carbon was 7.1 Å, which is far from optimal. For warfarin, the largest angle (144.1°) was recorded for ZEN-lactonase, but the distance of 5.7 Å also does not allow efficient catalysis to be expected. Thus, it was established that the larger coumarin molecules do not form catalytically competent complexes with any of the investigated enzymes.

In summary, under the strict geometric criterion (angle ~180°), only PON2 with coumarin displays parameters potentially favorable for hydrolytic opening of the lactone ring. The other small coumarins (DAMC, DEAMC) have no suitable candidate catalysts among the investigated enzymes, since in no complex are a short distance and an angle close to 180° realized simultaneously.

This indicates that the ability of enzymes to bind coumarins in the active site is in itself insufficient for catalysis to occur and that strict orientation of the substrate, ensuring a linear trajectory of nucleophilic attack, is required.

### 2.4. Assessment of the Potential Enzymatic Hydrolysis of Statins In Silico

**Figure 9:**
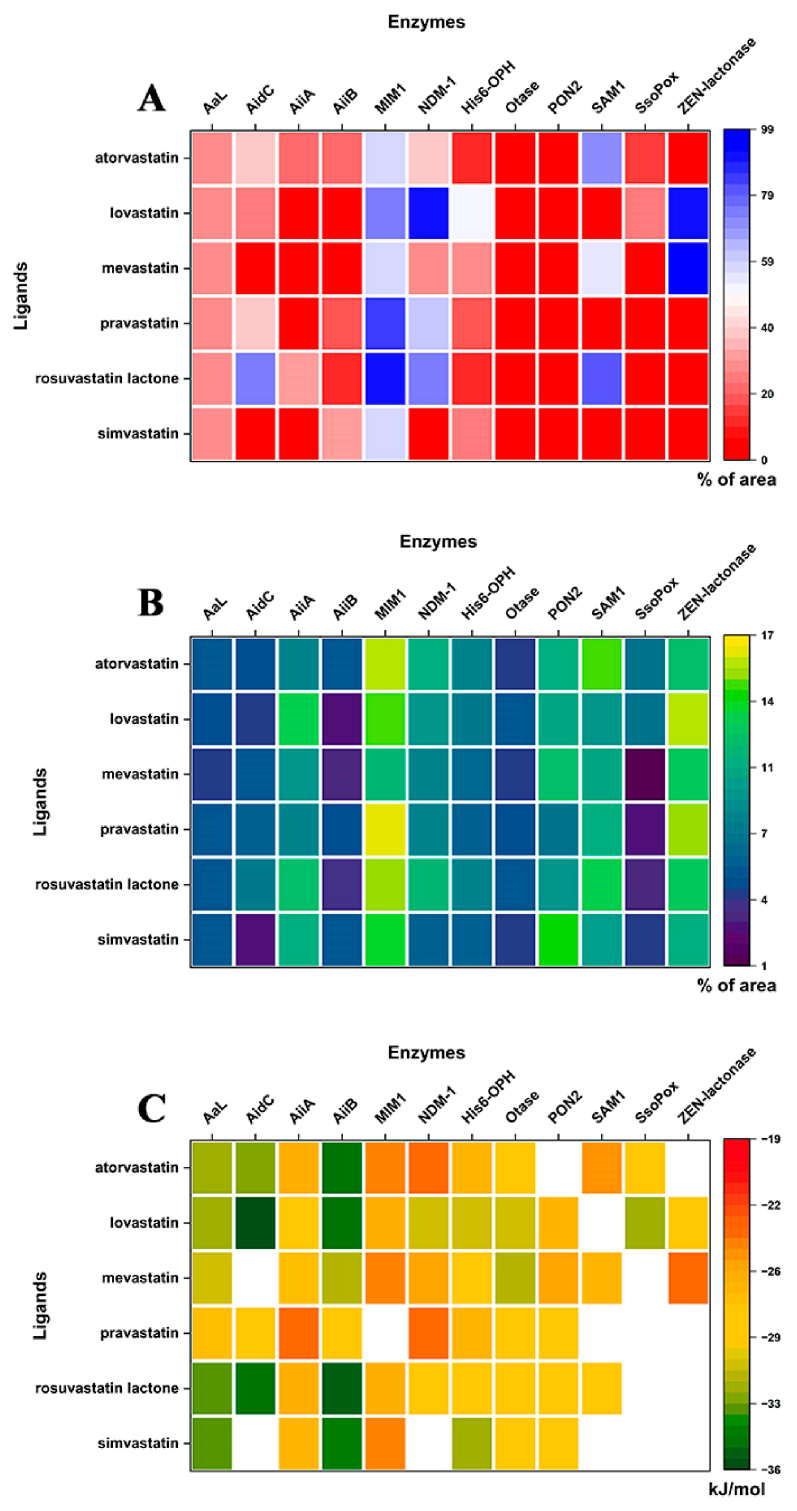
Surface area (%) occupied by statin molecules on the enzyme surface in the region of their active sites (**A**), or on the entire enzyme surface (**B**). The surface area of the enzyme active sites themselves (**A**) or the entire enzyme surface (**B**) was taken as 100%. Also shown are the binding affinity (kJ/mol) of statin molecules with the enzyme surface in the active site region (**C**).

**Figure 10:**
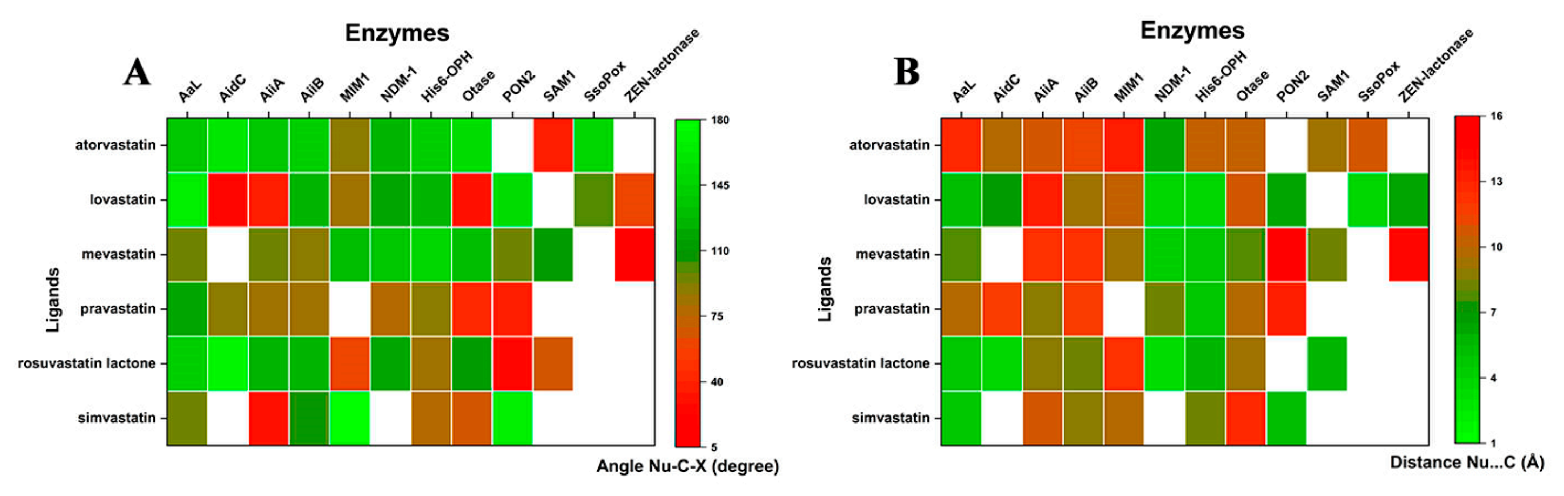
Calculated nucleophilic attack parameters for enzyme–statin complexes. Shown are (**A**) attack angles (degrees) defining the trajectory of substrate approach to the reaction center (Nu–C–X, where X = O, N for lactone and lactam, respectively), and (**B**) distances from the nucleophile to the electrophilic carbonyl carbon of the lactone/lactam ring (it is marked as Nu…C). The most favorable values of the geometric parameters for the catalytic reaction to proceed are indicated in green, and the least favorable are shown in red. Cases in which the ligands are distant from the enzyme active site are uncolored.

Interaction models of the 12 enzymes with six statins were simulated in silico (Figure S1–S12), and the obtained results are presented in Figure 9 and Figure 10.

A significant proportion of the active site was occupied in the MβLs (MIM-1, NDM-1, and SAM-1), as well as in ZEN-lactonase and His_6_-OPH. The highest values were recorded for ZEN-lactonase upon interaction with mevastatin (98.8%) and lovastatin (89.5%), for MIM-1 with rosuvastatin (90.2%) and pravastatin (85.4%), and for NDM-1 with lovastatin (89.4%) and rosuvastatin (74.5%). The enzyme His_6_-OPH occupied up to 52.3% of the active site with lovastatin.

AaL, AiiB, and SsoPox showed moderate occupancy (16–33%), whereas AidC, OTase, and PON2 practically did not accumulate statins in the active-site region, with the exception of AidC with rosuvastatin (75.1%) and atorvastatin (39.3%).

The highest affinity was observed for AidC with lovastatin (−36.0 kJ/mol) and rosuvastatin (−34.6 kJ/mol), for AiiB with rosuvastatin (−35.5 kJ/mol) and atorvastatin (−34.9 kJ/mol), and for AaL with simvastatin (−32.8 kJ/mol) and rosuvastatin (−32.9 kJ/mol). His_6_-OPH exhibited high affinity for simvastatin (−31.6 kJ/mol) and lovastatin (−31.0 kJ/mol). All these values fall within the strong binding category (≤−28 kJ/mol). The classical MβLs (MIM-1, SAM-1) possessed considerably lower affinity for all statins (from −24.1 to −27.8 kJ/mol), which corresponds to weak binding according to the same criteria [24,25].

Analysis of the distances and nucleophilic attack angles, carried out taking into account that efficient hydrolysis of the lactone ring requires an angle close to 180° (linear attack along the C–O bond), revealed a very limited number of combinations satisfying this criterion (Figure 10).

The only pair in which a short distance (3.9 Å) and an angle close to linear (168.7°) are simultaneously realized is the AidC–rosuvastatin lactone complex. For lovastatin, an angle of 166.2° in the complex with AaL is achieved at a distance of 5.5 Å, which somewhat exceeds the optimal range; nevertheless, this combination merits attention as potentially active.

The enzymes His_6_-OPH with mevastatin (4.5 Å, 150.4°) and PON2 with simvastatin (5.2 Å, 167.3°) displayed moderate deviations in distance, but the attack angles were sufficiently close to the required value, making these enzyme–ligand pairs candidates for further experimental verification. All other complexes, including those that exhibited high affinity or substantial active-site occupancy (e.g., MIM-1 with rosuvastatin, NDM-1 with lovastatin), do not provide a linear trajectory of nucleophilic attack: their angles lie in the range of 25–144°, which is incompatible with productive catalysis.

Thus, among the investigated enzymes, only AidC displays virtually ideal geometry for the hydrolysis of rosuvastatin lactone, whereas AaL, His_6_-OPH, and PON2 are of interest for lovastatin, mevastatin, and simvastatin, respectively.

### 2.5. Analysis of the Inhibition of Lactonases by the Investigated Lactone-Containing Compounds

The selection of enzymes and compounds for experimental validation was based on the molecular docking results: pairs exhibiting the most favorable predicted geometry and simultaneously representing different classes of compounds were selected. For experimental verification of the predicted inhibitory properties, the kinetic parameters of inhibition were determined for NDM-1 and His_6_-OPH by three representatives of different classes of pharmaceutical pollutants: azithromycin, warfarin, and simvastatin (Figure S13, Table 2).

All investigated compounds caused a decrease in the enzymatic reaction rate; however, the mechanism of inhibition differed depending on the nature of the enzyme and the investigated ligand.

For His_6_-OPH, warfarin and simvastatin exhibited a mixed type of inhibition with a pronounced uncompetitive component: the inhibition constants for V_max_ (K_ii_) were 169 and 1.4 μM, respectively, which are orders of magnitude lower than the Kis values characterizing the effect on the apparent substrate affinity. This indicates preferential binding of the inhibitor to the enzyme–substrate complex. Azithromycin with respect to His_6_-OPH, by contrast, displayed mixed inhibition with a predominance of the competitive component (K_is_ = 20.6 μM, K_ii_ = 93.9 μM), i.e., it mainly competed with the substrate for the active site.

For NDM-1, azithromycin also acted as a mixed inhibitor with dominance of the uncompetitive mechanism (K_ii_ = 3.4 μM, K_is_ = 19.7 μM). Simvastatin and warfarin in the case of NDM-1 exhibited mixed inhibition with relatively close K_ii_ and K_is_ values (simvastatin: 18.1 and 27.3 μM; warfarin: 63 and 61.6 μM), which approximates a non-competitive type and suggests roughly equal affinity for the free enzyme and the ES complex.

The obtained results indicate that the presence of macrolides, coumarins, and statins in wastewater can not only create an additional toxic load but also suppress the activity of enzymes used for bioremediation. In particular, His_6_-OPH, known for its effectiveness in the detoxification of organophosphorus pesticides and mycotoxins [26,27], may be substantially reduced in the presence of simvastatin and azithromycin, which act as mixed inhibitors with micromolar affinity.

Thus, when developing enzymatic systems for wastewater treatment, it is necessary to take into account the possibility of inhibition of key biocatalysts by components of complex multicomponent mixtures of pharmaceutical pollutants.

**Figure 11:**
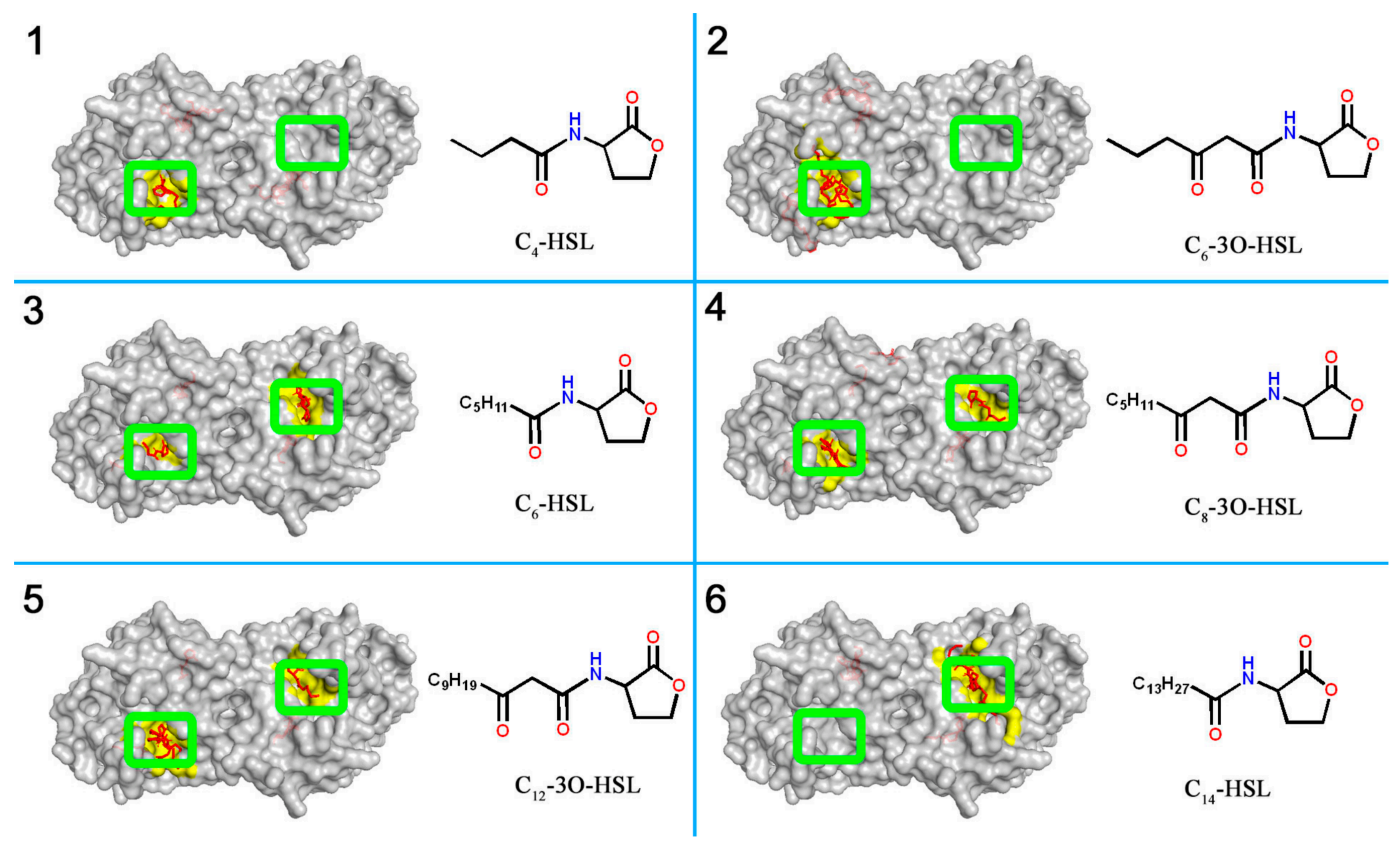
The location of bacterial lactone-containing QS molecules (1—C_4_-HSL, 2—C_6_-3O-HSL, 3—C_6_-HSL, 4—C_8_-3O-HSL, 5—C_12_-3O-HSL, 6—C_14_-HSL) on the surface of the NDM-1 dimer. The surface of the NDM-1 dimer molecule is indicated by a translucent gray color. The atoms located within 4 Å of any atom of the A-HSLs and the corresponding molecular surface of the enzyme are colored yellow. The entrances to the active sites of NDM-1 are marked with green rectangles. The structural formula of the corresponding ligand is shown adjacent.

In silico, a study of the interactions of NDM-1 lactamase with various A-HSLs molecules produced by Gram-negative bacteria and often present in water samples after their biological treatment [28,29] revealed the possible presence of almost all ligands in the active center of the enzyme (Figure 11, Table 3).

**Figure 12:**
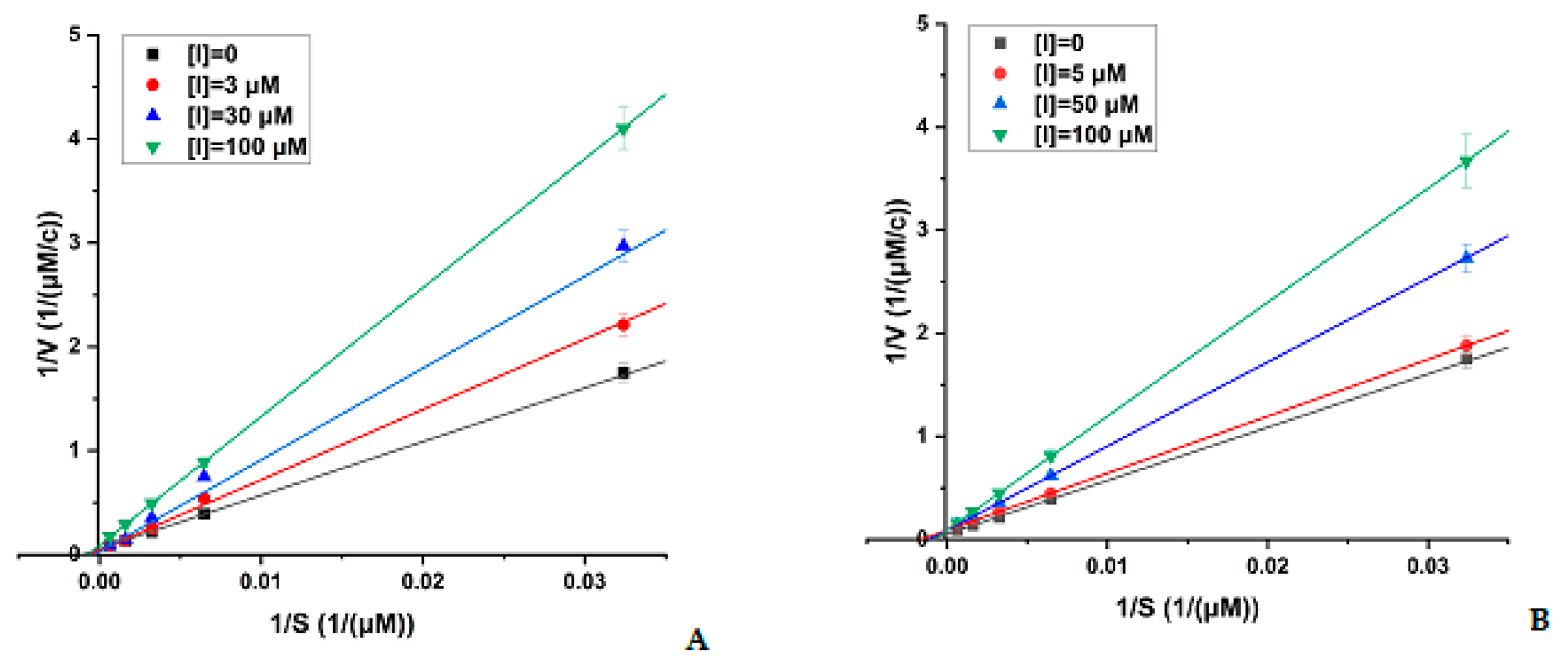
Dependence of the initial rate of the enzymatic reaction on the substrate concentration in the Lineweaver–Burk coordinates for NDM-1 in the presence of C_6_-(3O)-HSL (**A**) and C_4_-HSL (**B**).

According to the interaction affinity values (Table 3), sufficiently stable interactions between NDM-1 and the lactone-containing QS molecules such as C_4_-HSL and C_6_-3O-HSL could be observed. The affinity of both compounds for NDM-1 appeared to be the highest among the other A-HSLs. Therefore, the possible catalytic reactions involving NDM-1 and the two above-mentioned A-HSLs were investigated in the next stage of the work. The results are presented in Figure 12.

It was established that neither compound was hydrolyzed by the enzyme, but competitive inhibition of NDM-1 was revealed in experiments with varied concentrations of C_4_-HSL and C_6_-3O-HSL. According to the obtained data, the competitive inhibition constants (K_i_) were 76 µM and 90 µM for C_6_-(3O)-HSL (A) and C_4_-HSL (B), respectively.

## 3. Discussion

The systematic molecular modeling of 468 enzyme–ligand combinations, followed by analysis not only of binding affinity but also of key geometric parameters—the distance from the nucleophile to the carbonyl carbon atom of the lactone/lactam ring and the nucleophilic attack angle—has made it possible, for the first time, to assess the limits of substrate specificity of 12 hydrolases towards 39 pharmaceutical pollutants containing cyclic ether and amide structures. The obtained results demonstrate that neither high affinity nor substantial active-site occupancy is always sufficient for predicting catalytic competence; the fine geometry of substrate binding is of decisive importance, ensuring the approach of the reaction centers to the distance required for nucleophilic attack and the optimal trajectory of that attack.

For β-lactam antibiotics, the in silico predicted catalytic competence of the MβLs studied here (NDM-1, MIM-1, SAM-1) is substrate-dependent rather than uniformly limited by geometric criteria. Among these enzymes, NDM-1 is distinguished by the broadest substrate specificity; therefore, its interaction with β-lactams cannot be reduced to a single restrictive distance/angle threshold. For some β-lactam scaffolds, the calculated distance from the β-lactam carbonyl carbon to the metal ions coordinating the nucleophilic water molecule and the nucleophilic attack angle fall within ranges compatible with productive catalysis, whereas for other scaffolds high affinity and substantial active-site occupancy are not accompanied by an optimal attack trajectory. Thus, even for NDM-1, high affinity or substantial active-site occupancy alone is not always sufficient for catalytic competence; the fine geometry of substrate binding remains decisive.

For MIM-1 and SAM-1, the distances from the carbonyl carbon atom of the β-lactam ring to the metal ions coordinating the nucleophilic water molecule did not exceed 4–5 Å in most cases, with attack angles of 90–130°. This indicates that the ligands and the enzyme active sites come into proximity, yet the nucleophilic attack angle does not always allow a productive catalytic outcome to be expected.

Of particular note are the bifunctional enzymes MIM-1 and SAM-1, which, as has been shown previously, also possess lactonase activity towards N-acyl homoserine lactones [13]. The results obtained indicate that, in the presence of β-lactam antibiotics, it is the β-lactamase activity of these enzymes that should dominate, making them attractive candidates for the targeted degradation of penicillins and cephalosporins in wastewater. Table 4 presents the levels of contamination of wastewater and surface water bodies located in different geographical regions of the world by various pharmaceutical compounds that contain lactam or lactone rings in their structure ( Figure 1 ).

According to the data presented, it is precisely β-lactam antibiotics that are widely prevalent among the pharmaceutical pollutants found in various wastewater samples. Moreover, the data presented in Table 4 [3,4,5,30,31,32,33,34,35,36,37,38,39,40,41] confirm the broad occurrence of pharmaceutical pollutants in different wastewaters and the relevance of addressing the issues associated with them that are raised in this work.

Lactonases belonging to the same MβL superfamily (AaL, AidC, AiiA, AiiB), despite structural similarity, failed to provide ‘productive’ geometry for the majority of β-lactams. Although the attack angles sometimes fell within the range favorable for β-lactams (from 140° to 180°), the distances to the carbonyl carbon atom of the lactam ring regularly exceeded 7–9 Å. This is explained by the evolutionary adaptation of their active sites to larger and more flexible lactone substrates, A-HSLs, rather than to the strained β-lactam ring. ZEN-lactonase, by contrast, actively bound cephalosporins in the active site, but the highly unfavorable attack angles (less than 50°) rule out the possibility of catalysis, which once again underscores the necessity of a combined analysis of occupancy and geometry.

For macrolide antibiotics, none of the investigated enzymes achieved a combination of a short distance and an angle close to 180°. The minimum measured distances (5.3–5.7 Å) were observed for SAM-1 with erythromycin and clarithromycin, but the attack angles (122° and 88°, respectively) are far from those required. Even in the few cases where the nucleophilic attack angle value approached the optimum (AiiA–roxithromycin 174.5°), the distance exceeded 13 Å. These results are in good agreement with the known resistance of macrolides to a broad spectrum of hydrolases and explain the necessity of using specialized esterases, such as erythromycin esterase, for their degradation [21]. Consequently, the detoxification of macrolides in the environment will require either the search for new enzymes or the directed evolution of existing hydrolases with the aim of radically remodeling the active site.

In the case of coumarins, a clear dependence was found between the size of the substituents and the ability to form productive complexes. Small coumarins (coumarin, DAMC, DEAMC) found several suitable partners: AaL, AidC, NDM-1, MIM-1, and SAM-1 consistently provided distances of 3.0–4.6 Å at attack angles of 92–145°. It is noteworthy that His_6_-OPH and PON2 also emerged as candidates for these substrates, extending their known lactonase activity [14,16,17] to aromatic lactones.

Warfarin and C47T1, which carry bulky substituents, proved practically inaccessible for hydrolysis: even with high affinity (e.g., AaL/warfarin, –31.2 kJ/mol), the distances to the reactive carbon were 6.1–9.1 Å. This may suggest that binding occurs predominantly through hydrophobic contacts of the aromatic system rather than through proper orientation of the lactone ring within the catalytic site; however, this remains a hypothesis based on visual inspection of the docking poses and requires further quantitative analysis of the contact contributions. An interesting exception is MIM-1 with warfarin (4.5 Å, 75.6°); however, the attack angle here is far from ideal, and the probability of hydrolysis remains low.

The most unexpected and practically significant results were obtained for statins. In this study, several combinations of statins with enzymes whose active-site geometry is practically ideal for hydrolysis of the lactone ring were identified. Lovastatin in complexes with His_6_-OPH (3.7 Å, 121.7°), SAM-1 (3.5 Å, 99.4°), and NDM-1 (3.6 Å, 118.9°); rosuvastatin lactone with NDM-1 (3.4 Å, 120.5°); and simvastatin with AaL (4.7 Å, 94.1°) display parameters characteristic of productive binding.

His_6_-OPH deserves particular attention, as it was previously known as an efficient lactonase towards A-HSLs and organophosphorus compounds [14]. The obtained data predict that the substrate spectrum of this enzyme may be expanded to include statins in the lactone form. This opens the way to the development of biocatalytic systems for the final polishing treatment of wastewater contaminated with cholesterol-lowering drugs, especially since such contaminants are regularly detected in water bodies [39,40,41].

Summarizing all the obtained data by means of heatmaps (Figure 3, Figure 4, Figure 5, Figure 6, Figure 7, Figure 8, Figure 9 and Figure 10), constructed from the aggregate of established distances and angles in intermolecular interactions, revealed two principal functional clusters. The first unites the classical MβLs (NDM-1, MIM-1, SAM-1) with β-lactam antibiotics and, partially, with small coumarins and statins. The second cluster is formed around the enzymes His_6_-OPH and PON2, which showed complementarity to coumarins and statins, and, in individual cases, to cephalosporins. The lactonases AaL, AidC, AiiA, AiiB occupy an intermediate position, exhibiting moderate activity only towards coumarins.

In analyzing the obtained data, it is important to highlight certain limitations of the approach used to investigate intermolecular interactions. Molecular docking performed with AutoDock Vina does not account for the dynamic flexibility of the protein, the influence of ionic strength, or possible conformational rearrangements of the active site that could bring the reacting groups closer together after the initial binding of the molecules. Furthermore, the criteria of ‘favorable geometry’ (distance ~3–5 Å, nucleophilic attack angle close to 180°) are empirical and may vary for different enzyme classes [23,42].

Therefore, all the predictions made require mandatory experimental verification by determining the kinetic parameters of hydrolysis in vitro. Nevertheless, the high predictive capacity of an analogous approach, demonstrated previously for His_6_-OPH and MIM/SAM lactamases [13,14], allows the obtained results to be regarded as a reliable basis for the selection of promising enzyme biocatalysts, their substrates, or inhibitors.

Experimental verification of the inhibitory capacity of azithromycin, warfarin, and simvastatin towards NDM-1 and His_6_-OPH showed that all three compounds indeed suppress the activity of both enzymes, albeit with different mechanisms and efficiencies. For His_6_-OPH, simvastatin proved to be the most effective inhibitor (K_ii_ ≈ 1.4 μM) with a predominance of the uncompetitive component. This means that in real wastewater, the enzyme His_6_-OPH, known for its ability to hydrolyze toxic organophosphorus compounds [14], may noticeably lose activity in the presence of even low concentrations of simvastatin.

Among the investigated inhibitors, azithromycin showed the greatest effectiveness towards NDM-1 (K_ii_ ≈ 3.4 μM). The inhibition mechanism was of a mixed type with a predominance of the uncompetitive component. The discovered inhibition of NDM-1 by macrolides is of particular practical interest, as it may point to an additional mechanism of antimicrobial action for compounds of this class on bacterial cells. Macrolides are traditionally considered as inhibitors of protein synthesis that bind to the 50S ribosomal subunit [43]. However, their ability, revealed in this work, to suppress the activity of the NDM-1 lactamase, and at micromolar concentrations, suggests that macrolides may weaken the β-lactamase defense of bacteria, rendering them more vulnerable to β-lactam antibiotics. This assumption is in good agreement with the fact that acquired resistance to macrolides generally spreads more slowly and is less pronounced than resistance to β-lactams.

This result highlights the importance of taking into account cross-interactions of enzymes and pharmaceutical pollutants when developing biocatalytic water treatment systems.

The inhibition of NDM-1 by macrolides, revealed in this work, can be taken into account as a theoretical basis of those positive action effects, which were multiply confirmed for the combinations of the macrolides with β-lactams in their use in practice with patients [44,45,46,47].

Comparison of the obtained inhibition constants for NDM-1 with the known characteristics of classical β-lactamase inhibitors that are active against MβLs shows that azithromycin, in terms of K_ii_ (3.4 μM), is markedly less potent than xeruborbactam (K_i_ = 7.5 nM) and taniborbactam (K_i_ = 4.5 nM) but substantially more effective than vaborbactam, avibactam, relebactam, and durlobactam, which exhibit no significant inhibition of NDM-1 (K_i_ > 160 μM) [48]. At the same time, the K_is_ of azithromycin (19.7 μM) is noticeably higher, indicating preferential binding of the inhibitor to the enzyme–substrate complex rather than to the free enzyme. It is important to note that azithromycin is a macrolide antibiotic rather than a classical β-lactamase inhibitor, and its interaction with NDM-1 may therefore involve a different mechanism than that of boronate- or diazabicyclooctane-based inhibitors.

Simvastatin (K_ii_ 18.1 μM) and warfarin (K_ii_ 63 μM) are inferior in affinity to classical MβL inhibitors; however, their inhibition constants are in the micromolar concentration range, which attests to their significant practical potential as lactamase inhibitors. It is important to emphasize that the investigated compounds are not structural analogues of β-lactams, and the discovered ability to suppress NDM-1 activity points to the possibility of their binding to the enzyme through previously undescribed mechanisms. The present study was performed with purified enzymes and molecular modeling. No bacterial cultures, MIC determinations, checkerboard assays, time-kill experiments, periplasmic or in vivo studies were conducted. Therefore, the therapeutic implications discussed here are presumptive and should be tested experimentally.

The obtained data underscore an important practical aspect: when formulating enzyme compositions for wastewater treatment, it is necessary to take into account not only the catalytic efficiency of individual enzymes towards target substrates but also their sensitivity to inhibitors inevitably present in multicomponent mixtures of pharmaceutical pollutants. This is particularly relevant for His_6_-OPH, which is promising for application in the degradation of organophosphorus pesticides [26] and mycotoxins [27], but may be deactivated by statins and macrolides.

The concentrations of the pharmaceutical pollutants (Table 4) were found to be lower than the inhibition constants determined for the studied enzymes (Table 2); however, elevated concentrations of these pollutants may occur in activated sludge due to their accumulation during the wastewater treatment process. Microbial cells in the sludge can enhance their resistance to the pollutants through QS mechanisms. Gram-negative bacteria produce AHLs, the accumulation of which does not pose a problem for most lactonases but can inhibit the activity of NDM-1, which is active against β-lactam pollutants in wastewater. Taking this result into account, a decrease in the efficiency of β-lactam biodegradation can be expected as the abundance of AHL-producing bacteria in the activated sludge of wastewater treatment plants increases. This may be particularly expected in the case of *Pseudomonas* cells and therefore should be monitored.

## 4. Materials and Methods

### 4.1. Materials

NDM-1 was purchased from RayBiotech, Inc. (Norcross, GA, USA). The His_6_-OPH enzyme was produced, purified and characterized by a published procedure [49]. Meropenem, paraoxon, azithromycin, warfarin and simvastatin were purchased from Sigma-Aldrich (Darmstadt, Germany).

### 4.2. Computational Methods

Three-dimensional structures of enzymes were obtained from the Protein Data Bank. The known crystallographic structure of OPH (PDB ID: 1QW7) was used to prepare the structure of His_6_-OPH, which was further modified by His_6_-tag as described previously [50].

The human PON2 protein sequence (Swiss-Prot id Q15165) [51] was obtained at the UniportKB (http://www.uniprot.org/). A template for modeling the 3-D structure of human PON2 was identified by BLASTP (http://blast.ncbi.nlm.nih.gov/Blast.cgi (accessed on 10 August 2026)) [52] search against the Protein Data Bank (PDB) with default search parameters. PON 1 (1v04) [53] was selected as template to model PON2. The template structure was downloaded from PDB (https://www.rcsb.org) [54]. Based on the known structure of recombinant paraoxonase-1, the structure of PON2 was predicted with the I-TASSER server, taking into account existing amino acid substitutions [55].

All ligand structures, including A-HSLs, were created using ChemBioDraw software (ver. 12.0, CambridgeSoft, Waltham, MA, USA), and then energies were minimized using ChemBio3D software v.14 with the MM2 force field, and structures in the PDB format were obtained. Then, AutoDockTools (as part of MGLTools ver. 1.5.7, available https://ccsb.scripps.edu/mgltools/, accessed on 28 January 2026) was used to obtain structures in PDBQT format from PDB structures with atomic charges calculated by the Gasteiger–Marsili method [56].

APBS (Adaptive Poisson-Boltzmann Solver ver. 1.4.2.1) and PDB2PQR (ver. 2.1.1) servers, available at http://www.poissonboltzmann.org/ (accessed on 10 August 2026), were used to calculate the charge distribution on the surfaces of enzymes [57].

AutoDockVina (ver. 1.1.2, available at http://vina.scripps.edu/, accessed on 18 August 2022), which includes several terms in its own scoring function (Gaussian, repulsion, hydrophobic, hydrogen bonding, and the number of rotatable bonds) was used for docking [58]. The coordinates of the grid-box centers and dimensions for all 12 enzymes are provided in Supplementary Table S1. Based on our previous studies, the best 8 poses with minimal energy were selected. The calculations were performed on a desktop computer equipped with an Intel Pentium Dual-Core CPU E5400 2.7 GHz and 3 GB of available memory. The binding affinity values obtained with AutoDock Vina (in kcal/mol) were converted to kJ/mol using a conversion factor of 4.184.

Calculated interaction models were visualized in the PyMOL Molecular Graphics System (ver. 1.7.6, Schrodinger Inc., New York, NY, USA). The “get_area” function was used to obtain the values of the area occupied on the enzyme surfaces.

Distances and angles between nucleophilic atoms in the active site of the enzyme and ‘labile’ atoms of ligands were measured and visualized with PyMOL Molecular Graphics System.

It should be noted that the standard AutoDock Vina scoring function does not include an explicit term for metal coordination, and no AD4-type zinc parameters were assigned. Because the catalytic nucleophile in the studied metalloenzymes is a metal-activated water molecule, the distance from the nucleophile to the carbonyl carbon of the lactone/lactam ring was assessed using the metal ion as the nearest coordinating atom. This approach provides an approximation of the nucleophile–carbonyl distance under the limitations of the docking protocol.

### 4.3. Study of Inhibition of Enzymes with Lactonase Activity

For in vitro experimental verification of the potential inhibitory effect of the compounds selected in silico (azithromycin, warfarin, and simvastatin) on NDM-1 and His_6_-OPH, kinetic studies were performed in the presence of increasing concentrations of the lactone-containing compounds.

The enzymatic activity of NDM-1 was determined spectrophotometrically using meropenem as the substrate (1–100 μM), hydrolyzed in 20 mM HEPES buffer (pH 8.0) containing 10 μM ZnSO_4_ in accordance with known procedure [59].

The activity of His_6_-OPH was determined by the hydrolysis of paraoxon (1–100 μM) in 100 mM carbonate buffer (pH 10.5) [57]. In all experiments, the initial reaction rate (V) was recorded by the change in absorbance at the corresponding wavelength (λ = 297 nm for meropenem; λ = 405 nm for p-nitrophenol produced from paraoxon) at 25 °C on an Agilent 8453 UV–visible spectroscopy system (Agilent Technology, Waldbronn, Germany). The reaction was initiated by the addition of the enzyme (final concentration 50 nM for NDM-1, 2.5 nM for His_6_-OPH), and the rate was determined from the linear portion of the kinetic curve.

A number of A-HSLs were used in experiments with NDM-1 to analyze the interactions of the lactone-containing compounds with the enzyme: N-butyryl-DL-homoserine lactone (C_4_-HSL), N-hexanoyl-L-homoserine lactone (C_6_-HSL), N-(3-oxo hexanoyl)-L-homoserine lactone (C_6_-3O-HSL), N-(3-oxooctanoyl)-L-homoserine lactone (C_8_-3O-HSL), N-(3-oxododecanoyl)-L-homoserine lactone (C_12_-3O-HSL) and N-tetradecanoyl-L-homoserine lactone (C_14_-HSL). Such A-HSLs are usually present in wastewater systems [7,28,29].

For each inhibitor, a series of solutions was prepared with fixed inhibitor concentrations [I] (including a control with [I] = 0). At each inhibitor concentration, the initial rates V were measured at at least four different substrate concentrations [S]. For each [I], a Lineweaver–Burk plot was constructed by linear regression, and the slope a and intercept b were determined. Thus, for each inhibitor, a set of (a, b) pairs was obtained as a function of [I].

If the dependences of a on [I] and b on [I] were linear, this indicated a mixed type of inhibition (including special cases). The inhibition constants characterizing the effect of the inhibitor on the apparent Michaelis constant (K_is_) and on the maximum reaction rate (K_ii_) were calculated based on the equations for mixed inhibition:

(1) $$a\left(\left[I\right]\right)=a_{0}\cdot \left(1+\frac{\left[I\right]}{K_{\mathrm{is}}}\right)$$

(2) $$b\left(\left[I\right]\right)=b_{0}\cdot \left(1+\frac{\left[I\right]}{K_{\mathrm{ii}}}\right)$$

where a_0_ and b_0_ are the slope and intercept in the absence of inhibitor, respectively. After linear approximation,

(3) a([I]) = a_0_ + α · [I]

(4) b([I]) = b_0_ + β · [I]

the constants were determined as

(5) *K_is_* = *a*_0_/α

(6) *K_ii_* = *b*_0_/*β*

A change in only one parameter indicated a competitive (b = const, a increases) or uncompetitive (a = const, b increases) mechanism. Proportional changes in a and b (a/a_0_ = b/b_0_) corresponded to non-competitive inhibition, where K_is_ = K_ii_. In all other cases, when both parameters changed non-proportionally, the inhibition type was classified as mixed.

The inhibition constants characterizing the effect on the slope of the plot (K_is_) and on the maximum reaction rate (K_ii_) were calculated using standard formulae, applying linear regression of a and b against [I]. Experiments were performed in three independent replicates.

### 4.4. Statistical Analyses

Statistical analysis was performed using SigmaPlot (ver. 12.5, Systat Software Inc., San Jose, CA, USA), and the data are presented as the mean values of three independent experiments (*n* = 3) ± standard deviation (±SD). Pairwise multiple comparisons were conducted using the paired *t*-test and one-way analysis of variance (ANOVA) followed by the Holm–Sidak method. A value of *p* < 0.05 was considered statistically significant.

## 5. Conclusions

Visualization of the results of the intermolecular interaction study for all enzyme/ligand combinations, presented in the form of heatmaps, allowed the identification of variants in which inhibition of the enzymes by the investigated pharmaceutical lactone-containing compounds is possible. Determination of in vitro inhibition constants revealed the influence of lactone-containing pharmaceutical compounds with different therapeutic applications on enzymes that are classical lactamases and lactonases.

According to the obtained results, computational screening allowed the identification of a set of highly specific enzyme–substrate pairs that collectively cover several classes of pharmaceutical pollutants. Although the classical MβLs (NDM-1, MIM-1, SAM-1) demonstrated binding to the majority of β-lactams, only a limited number of combinations satisfied the strict geometric criteria, thereby confirming the correctness of the bioinformatics approach applied in this work to assess potential catalytic processes between the investigated enzymes and pharmaceutical pollutants.

Owing to the employed approach, several biocatalyst variants with virtually ideal geometry for the entry of statins as substrates into the enzyme active site were identified for the first time; in particular, lactonase AidC appeared to be a suitable catalyst for rosuvastatin, and lactonase AaL appeared suitable for lovastatin. In the case of coumarins, PON2 proved to be the sole promising catalyst for their hydrolysis among the investigated enzymes. These identified enzyme/pharmaceutical pollutant combinations are of the greatest interest for further detailed experimental verification.

Subsequent experimental validation of the predicted catalytic activities, as well as optimization of biocatalysis conditions, may become decisive for the transition from computational models to practically realizable bioremediation technologies. At the same time, the revealed ability of individual lactone-containing compounds to act as effective inhibitors of the studied hydrolases points to the need for additional analysis of enzyme compatibility within biocatalytic systems designed for the treatment of real multicomponent wastewater. The obtained data can be applied as a rational basis for the design of new compositions comprising several enzymes whose action may be directed towards the removal of β-lactams, coumarins, and statins from wastewater.

## Figures and Tables

**Table 1:** Hydrolytic enzymes selected for investigating their activity towards lactone-containing molecules.

Enzyme, Origin	Metals in the Active Center	PDB ID	pH Range of Action	References
Lactonase AaL from *Alicyclobacillus acidoterrestris*	Zn^2+^	6cgz	8.0–8.5	[9]
Lactonase AidC from *Chryseobacterium* sp.	Zn^2+^	4zo2	6.5–7.5	[10]
Lactonase AiiA from *Bacillus thuringiensis*	Zn^2+^, Mg^2+^, Co^2+^	2a7m	7.0–8.5	[11]
Lactonase AiiB from *Agrobacterium tumefaciens*	Zn^2+^, Co^2+^	2r2d	6.5–7.5	[12]
Metallo β-lactamase MIM-1 from *Novosphingobium pentaromativorans*	Zn^2+^, Co^2+^, Cu^2+^, Mn^2+^, Ca^2+^	6auf	7.5–8.0	[13]
Metallo β-lactamase SAM-1 (or MIM-2) from *Simiduia agarivorans*		6mfi		[13]
Metallo β-lactamase NDM-1 from *Klebsiella* *pneumoniae*	Zn^2+^	5ypi	6.5–8.0	[14]
Hexahistidine-containing organophosphate hydrolase (His_6_-OPH) from *Pseudomonas diminuta*	Co^2+^	1qw7 (OPH)	7.0–11.0	[14]
Lactonase SsoPox from *Sulfolobus solfataricus*	Fe^2+^, Co^2+^	2vc7	7.5–8	[15]
Serum paraoxonasa (PON2)	Ca^2+^	1v04 (PON1)	6.5	[16,17]
Zearalenone (ZEN-lactonase) from *Clonostachys rosea*	-	3wzl	6–8	[18]
Ochratoxinase (OTase) from *Aspergillus niger*	Zn^2+^	4c5y	6	[19]

**Table 2:** Inhibition constants characterizing the effect of azithromycin, warfarin, and simvastatin on the apparent Michaelis constant (K_is_) and on the maximum reaction rate (K_ii_) of the reaction catalyzed by the NDM-1 or His_6_-OPH.

Pharmaceutical Compound	NDM-1	His_6_-OPH
Azithromycin	K_ii_ ≈ 3.4 μM K_is_ ≈ 19.7 μM	K_ii_ ≈ 93.9 μM K_is_ ≈ 20.6 μM
Simvastatin	K_ii_ ≈ 18.1 μM K_is_ ≈ 27.3 μM	K_ii_ ≈ 1.4 μM K_is_ ≈ 8.1 μM
Warfarin	K_ii_ ≈ 63 μM K_is_ ≈ 61.6 μM	K_ii_ ≈ 169 μM K_is_ ≈ 1334 μM

**Table 3:** The area (%) occupied by bacterial A-HSLs in the region of the active sites of the NDM-1 dimer or on its entire surface, and the affinity of the enzyme’s interaction with ligands. The surface area of NDM-1 active sites is assumed to be 100% when calculating the area of ligand localization near them, and the total surface area of the NDM-1 dimer molecule is assumed to be 100% when calculating “in relation to total surface of enzyme molecule”.

#	Ligands	Near Active Site	In Relation to Total Surface of Enzyme Molecule	Affinity, kJ/mol
1	C_4_-HSL	45.0	5.1	−28.9 ± 0.4
2	C_6_-3O-HSL	44.8	6.4	−31.4 ± 1.3
3	C_6_-HSL	51.0	5.8	−18.0 ± 0.8
4	C_8_-3O-HSL	39.9	5.7	−19.2 ± 0.0
5	C_12_-3O-HSL	28.4	6.7	−18.4 ± 0.8
6	C_14_-HSL	31.6	7.9	−18.4 ± 0.8

**Table 4:** Level of contamination of wastewater by various pharmaceutical substances in different regions of the world.

Pharmaceutical Substance	Concentration Range, ng/L	Source	Area	References
**β-Lactam Antibiotics**				
Amoxicillin	<120	Wastewater	Italy, Roma	[30]
Penicillin G, Ampicillin	1051–2055 447–796	Wastewater treatment plant	Isfahan, Iran	[31]
Ceftazidime, Meropenem, Ampicillin, Cefixime, Amoxicillin	<60 <7 <2 <0.5 <50–1126	Urban stormwater runoff from residential areas; untreated and treated wastewater	Hoan Kiem Lake, West Lake and Yen So Lake, Hanoi, Vietnam	[32]
Cefotaxime	<18,000	Hospital wastewater and wastewater treatment plants	Ravi, Jhelum, Chenab, Indus, Kabul and Dor Rivers, Pakistan	[33]
Amoxicillin, Cefuroxime	145.0–659.0 <68	Surface water used for vegetable irrigation	Kumasi and Sunyani metropolises, Ghana	[34]
Amoxicillin Penicillin G	350–1020 20–310	Hospital wastewater treatment plants	Gonbad Kavous, Iran	[35]
Amoxicillin, Cefalexin, Penicillin G, Penicillin V	4.7–111	Anthropogenic activity	Taipu River, China	[3]
**Macrolides**				
Roxithromycin	4.0–48.1	River sediments	Zhangjiagang, China	[4]
Erythromycin	2.1–26.9	River sediments	Zhangjiagang, China	[4]
Azithromycin	13.4–2553.0	Wastewater treatment plant	Canale d’Aiedda Basin, Apulia Region, Southern Italy	[36]
Azithromycin Dihydroxyjosamycin Methylerythromycin Clarithromycin Josamycin	1220 21.5 13.5 21.3 13.8	Wastewater from a municipal treatment plant at the discharge point into the Hatoritsa River	Arkhangelsk, Russia	[5]
**Coumarins**				
7-dimethylamino-4-methylcoumarin	<800	Sediments	Dongjiang River basin, China	[37]
Chlorine derivatives of 7-hydroxycoumarin	<78	Drinking water after disinfection	Yangtze River, Nanjing, China	[38]
**Statins**				
Simvastatin	<50	Wastewater treatment plant	Guangdong Province, China	[39]
Atorvastatin	<14,620	Wastewater from hospitals and adjacent surface waters	Sri Lanka	[40]
Rosuvastatin	<64.9	7 rivers in Japan	Japan	[41]

## Data Availability

The original contributions presented in this study are included in the article’s Supplementary Materials. Further inquiries can be directed to the corresponding author.

## Supplementary Materials

The following supporting information can be downloaded at https://www.mdpi.com/article/10.3390/ijms27209061/s1.

## Abbreviations

The following abbreviations are used in this manuscript:

A-HSLs	N-acyl homoserine lactones
MβLs	metallo-β-lactamases
QS	Quorum Sensing
