# Supplementary material for: Actions of Pharmaceutical Lactones and Lactams on the Enzymes with Lactonase Activity: In Silico Prediction and In Vitro Study
Source: Int J Mol Sci. 2026 Oct 13;27(20):9061. doi: 10.3390/ijms27209061 (PMC13704903; doi:10.3390/ijms27209061)
Supplement: Supplementary file 1 [file ijms-27-09061-s001.zip › ijms-4556548-supplementary.pdf]

Article

Actions of Pharmaceutical Lactones and Lactams on the Enzymes with Lactonase Activity: In Silico Prediction and In Vitro Study

Elena Efremenko *, Maksim Domnin, Anastasia Sarapina and Aigul Zagidulina

Faculty of Chemistry, Lomonosov Moscow State University, Lenin Hills 1/3, Moscow 119991, Russia
* Correspondence: elena_efremenko@list.ru; Tel.: +7-(495)-939-3170

Supplementary materials

Table S1. Parameters of the grid boxes used for molecular docking.

No.	Enzyme	PDB ID	center x	center y	center z	size x	size y	size z
1	AaL	6cgz	-23	-43	18	60	60	58
2	AidC	4zo2	-24	13	-30	74	56	88
3	AiiA	2a7m	17	31	28	60	54	50
4	AiiB	2r2d	19	-24	-12	56	72	86
5	MIM-1	6auf	24	22	21	52	60	58
6	SAM-1 (MIM-2)	6mfi	59	86	21	54	52	50
7	NDM-1	5ypi	-49	-31	-71	52	80	60
8	His_6-OPH	1qw7 (OPH)	28	-18	28	66	90	60
9	SsoPox	2vc7	24	-15	5	54	58	100
10	PON2	1v04 (PON1)	63	62	63	60	76	54
11	ZEN-lactonase	3wzl	-17	19	10	50	50	50
12	OTase	4c5y	-28	69	-39	72	72	88

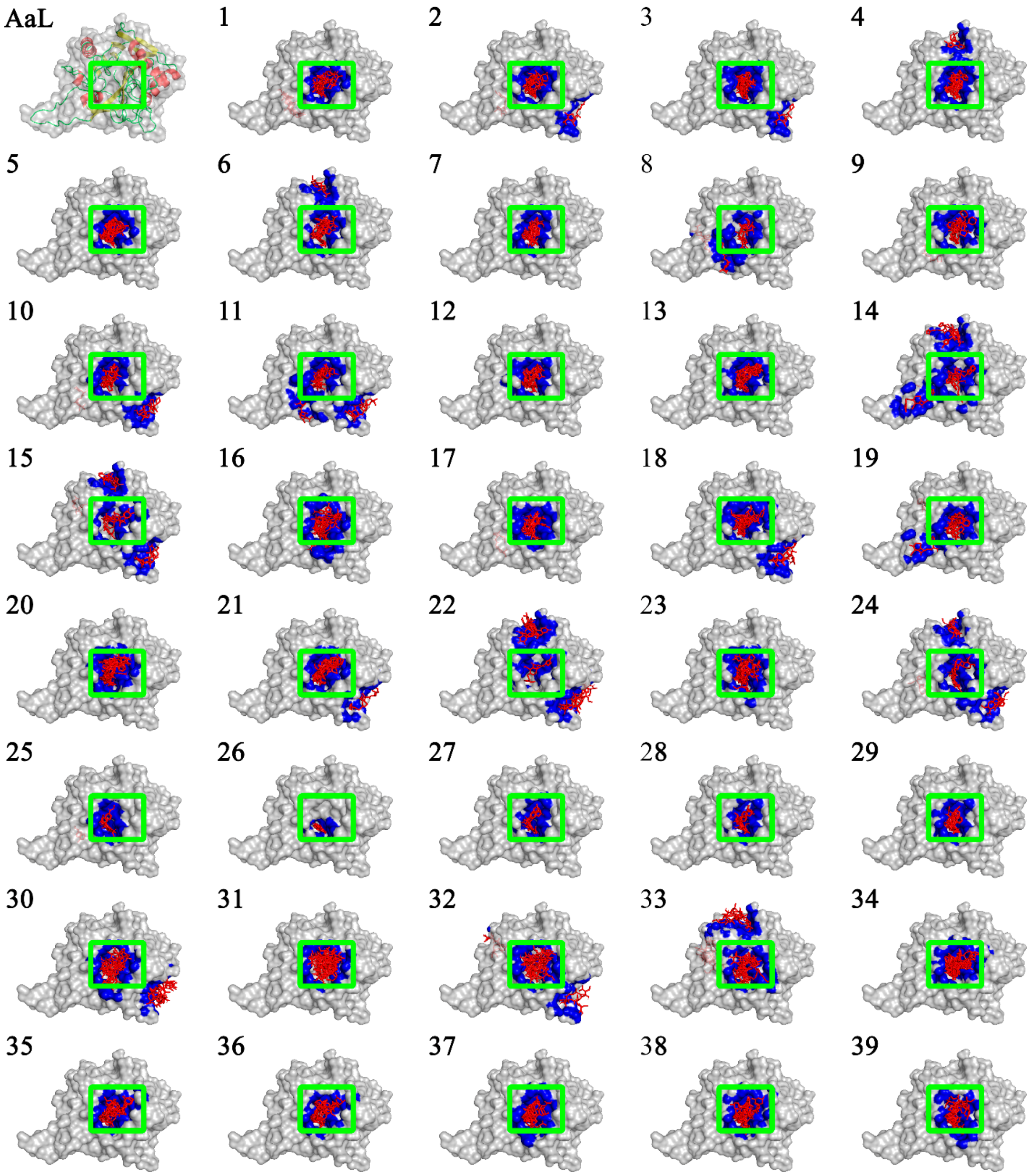

Figure S1. Localization of different ligand molecules (1 – meropenem, 2 – amoxicillin, 3 – ampicillin, 4 – cloxacillin, 5 – dicloxacillin, 6 – nafcillin, 7 – oxacillin, 8 – penicillin G, 9 – penicillin V, 10 – cefaclor, 11 – cefadroxil, 12 – cefalexin, 13 – cefamandole lithium, 14 – cefazolin, 15 – cefdinir, 16 – cefetamet pivoxyl, 17 – cefixime, 18 – cefotaxime, 19 – cefradine, 20 – ceftazidime, 21 – ceftiofur, 22 – ceftriaxone, 23 – cefuroxime axetil, 24 – desacetylcefotaxime, 25 – C47T1, 26 – coumarin, 27 – DAMC, 28 – DEAMC, 29 – warfarin, 30 – azithromycin, 31 – clarithromycin, 32 – erythromycin, 33 – roxithromycin, 34 – atorvastatin, 35 – lovastatin, 36 – mevastatin, 37 – pravastatin, 38 – rosuvastatin lactone, 39 – simvastatin) on the surface of enzyme **AaL**, obtained by computational studies of molecular interactions using molecular docking. The enzyme surface is shown in gray; ligand molecules are highlighted in red; blue color indicates the positions of ligand molecules within a distance of less than 4 Å from the enzyme surface. The catalytic center of the enzyme is outlined by a green rectangle.

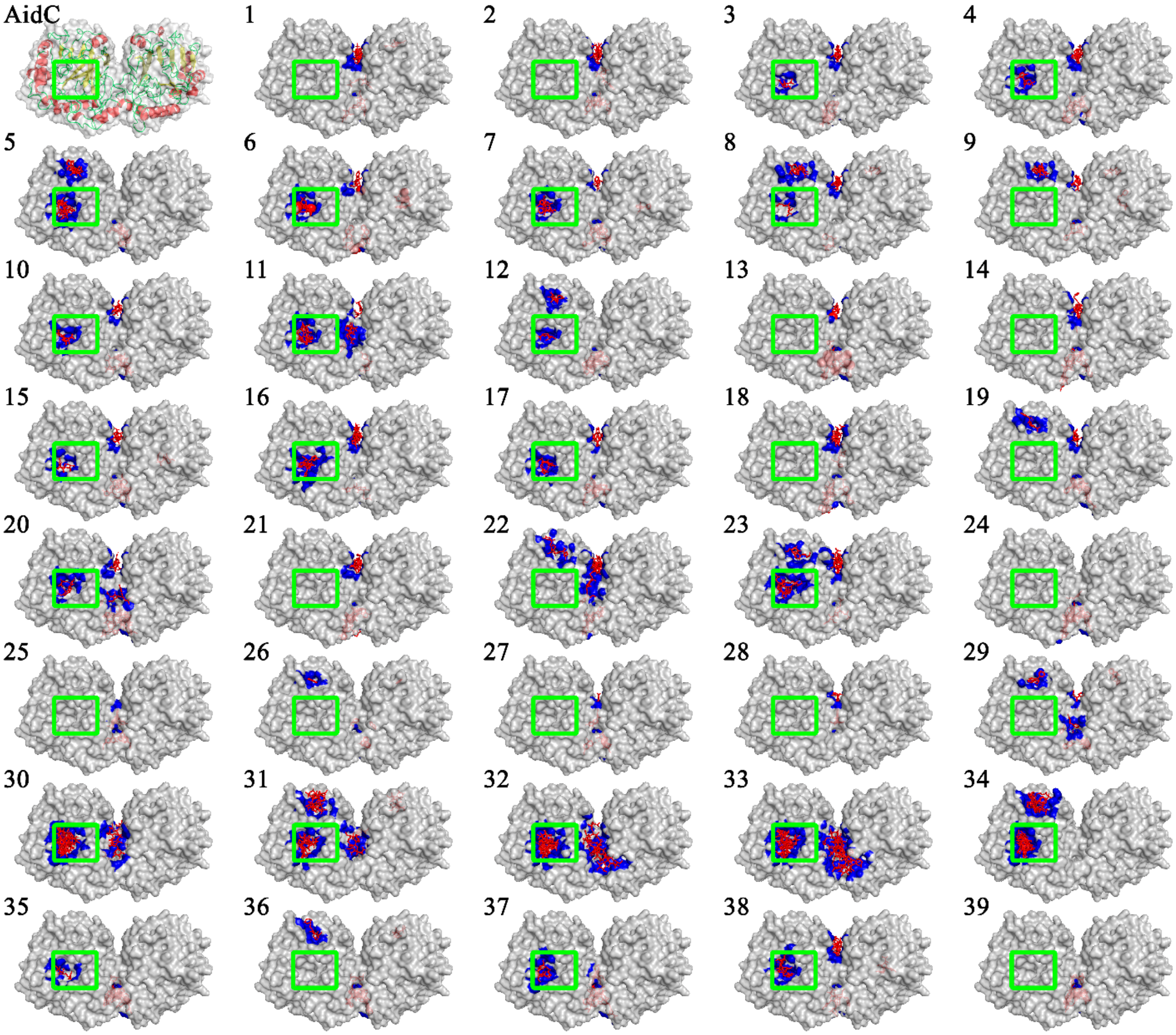

Figure S2. Localization of different ligand molecules (numbering 1–39 is given in the caption to Figure S1) on the surface of enzyme **AidC**, obtained by computational studies of molecular interactions using molecular docking: the enzyme surface is shown in gray; ligand molecules are highlighted in red; blue color indicates the positions of ligand molecules within a distance of less than 4 Å from the enzyme surface. The catalytic center of the enzyme is outlined by a green rectangle.

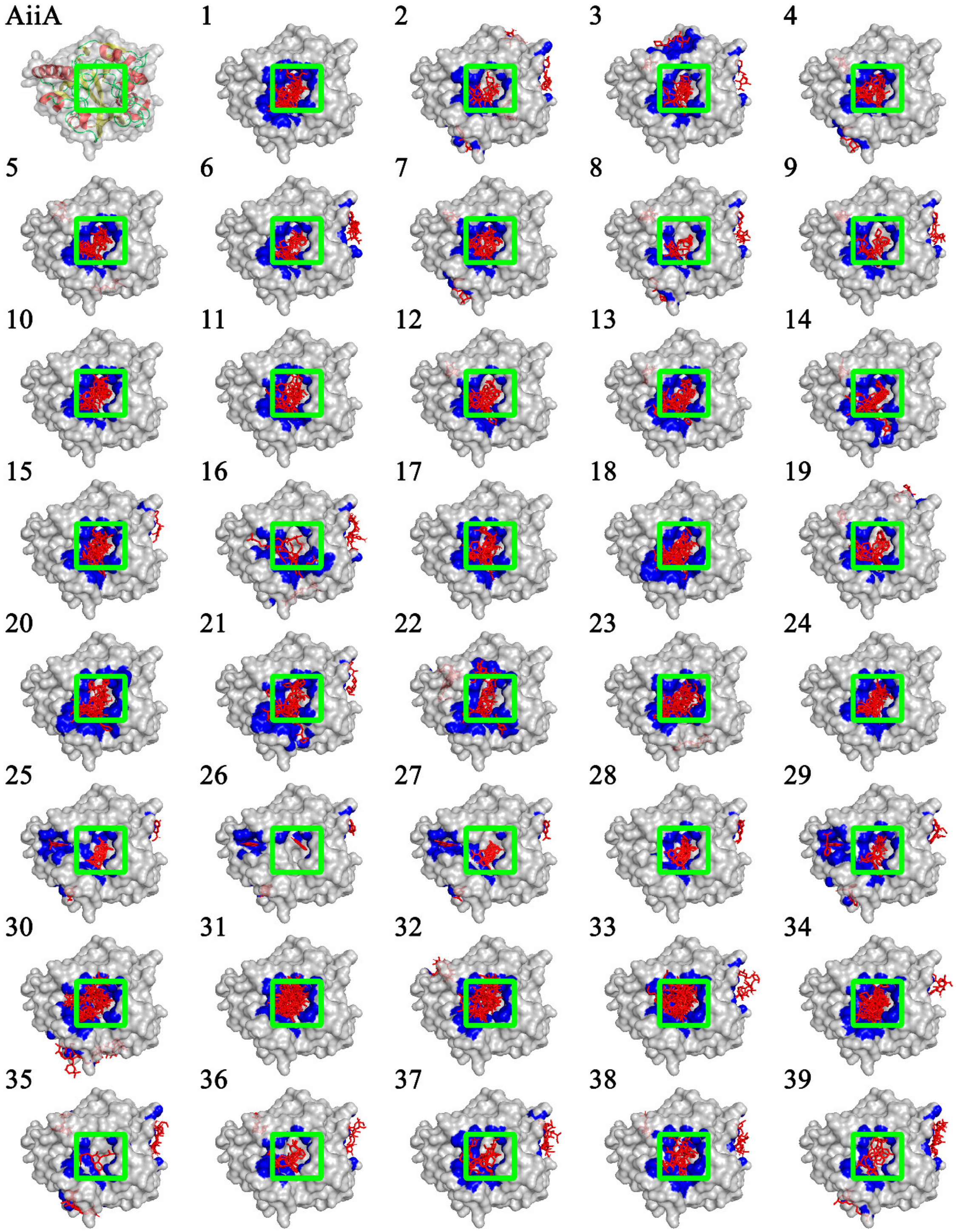

Figure S3. Localization of different ligand molecules (numbering 1–39 is given in the caption to Figure S1) on the surface of enzyme **AiiA**, obtained by computational studies of molecular interactions using molecular docking: the enzyme surface is shown in gray; ligand molecules are highlighted in red; blue color indicates the positions of ligand molecules within a distance of less than 4 Å from the enzyme surface. The catalytic center of the enzyme is outlined by a green rectangle.

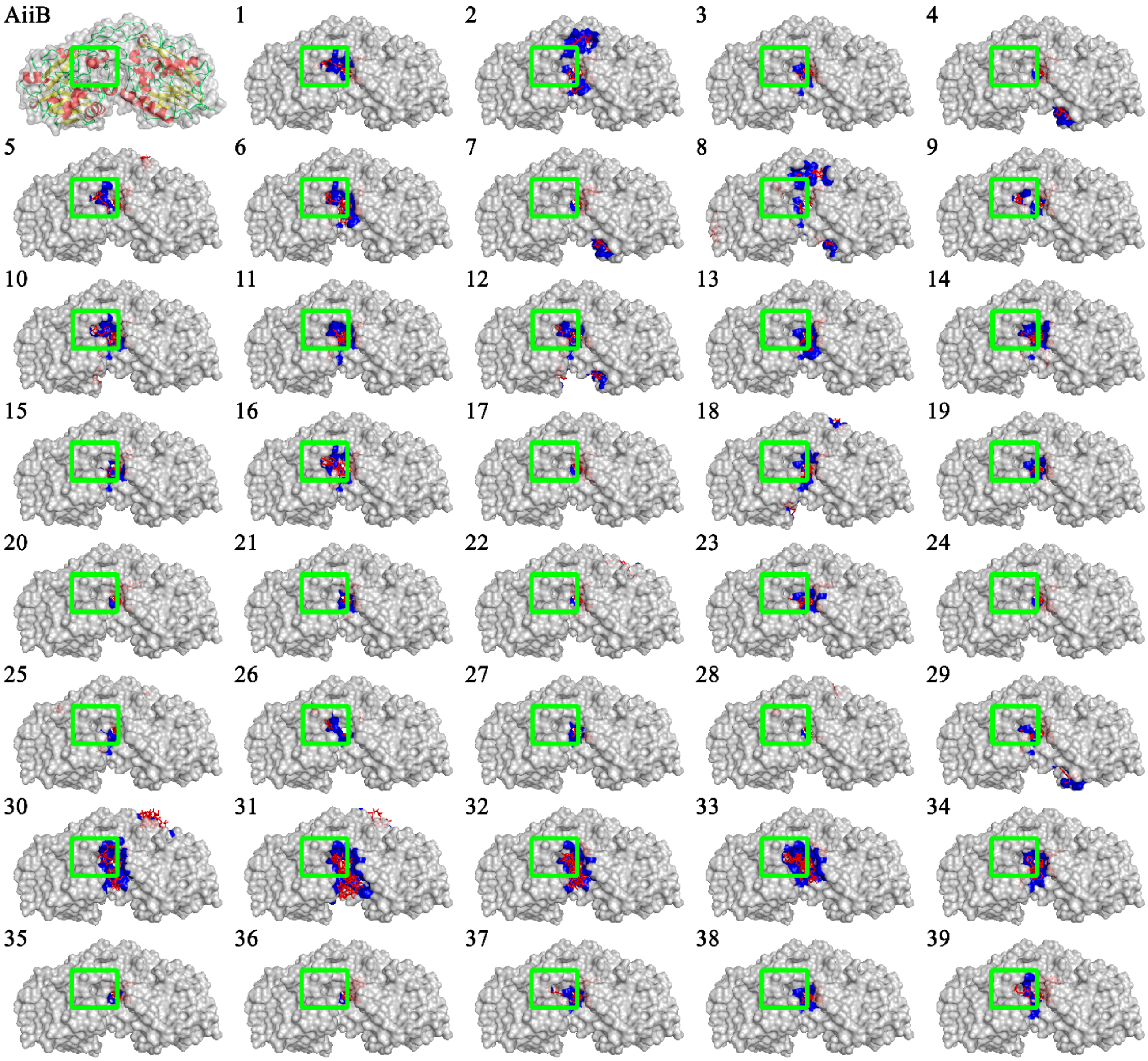

Figure S4. Localization of different ligand molecules (numbering 1–39 is given in the caption to Figure S1) on the surface of enzyme **AiiB**, obtained by computational studies of molecular interactions using molecular docking: the enzyme surface is shown in gray; ligand molecules are highlighted in red; blue color indicates the positions of ligand molecules within a distance of less than 4 Å from the enzyme surface. The catalytic center of the enzyme is outlined by a green rectangle.

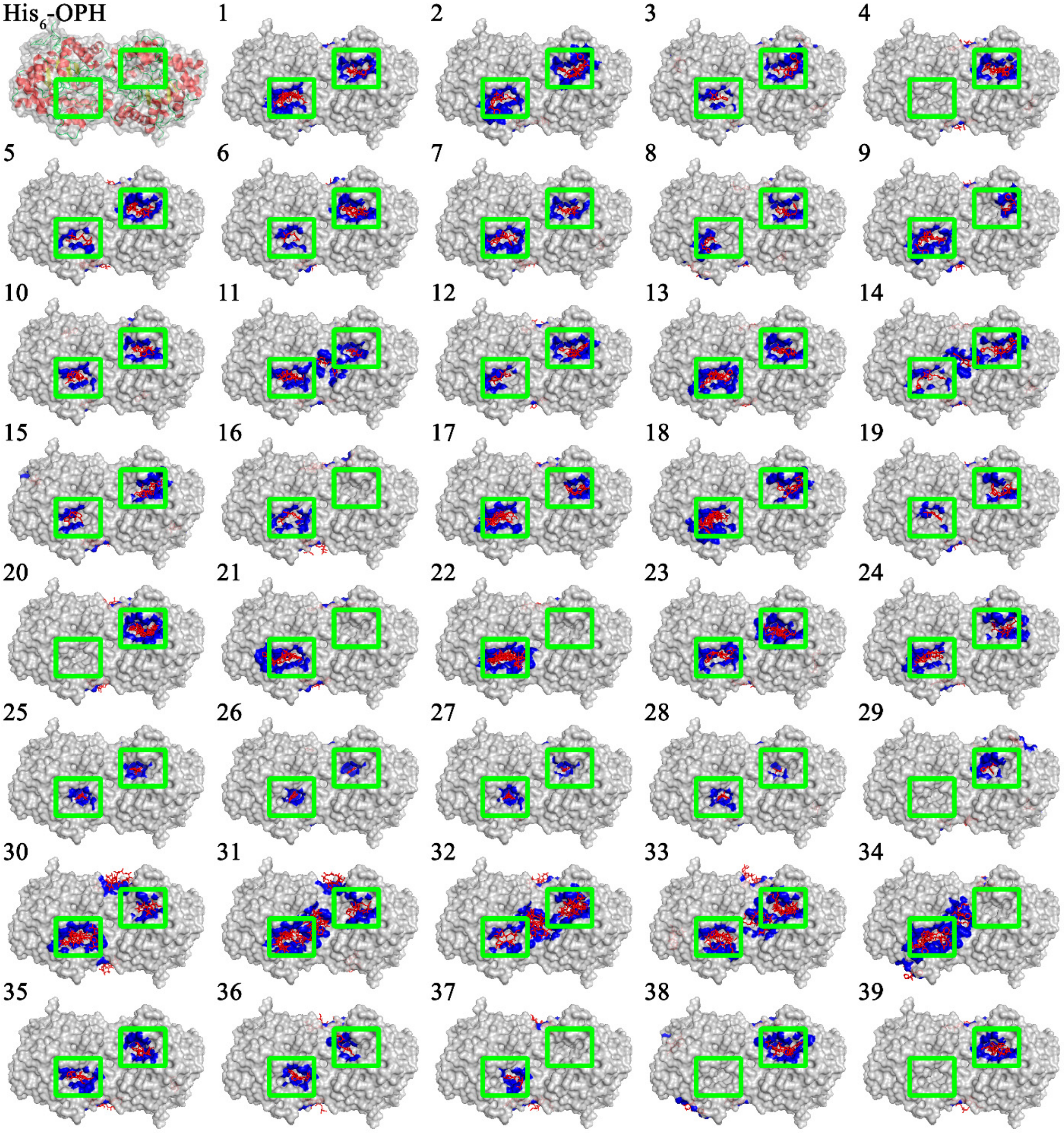

Figure S5. Localization of different ligand molecules (numbering 1–39 is given in the caption to Figure S1) on the surface of enzyme **His₆-OPH**, obtained by computational studies of molecular interactions using molecular docking: the enzyme surface is shown in gray; ligand molecules are highlighted in red; blue color indicates the positions of ligand molecules within a distance of less than 4 Å from the enzyme surface. The catalytic center of the enzyme is outlined by a green rectangle.

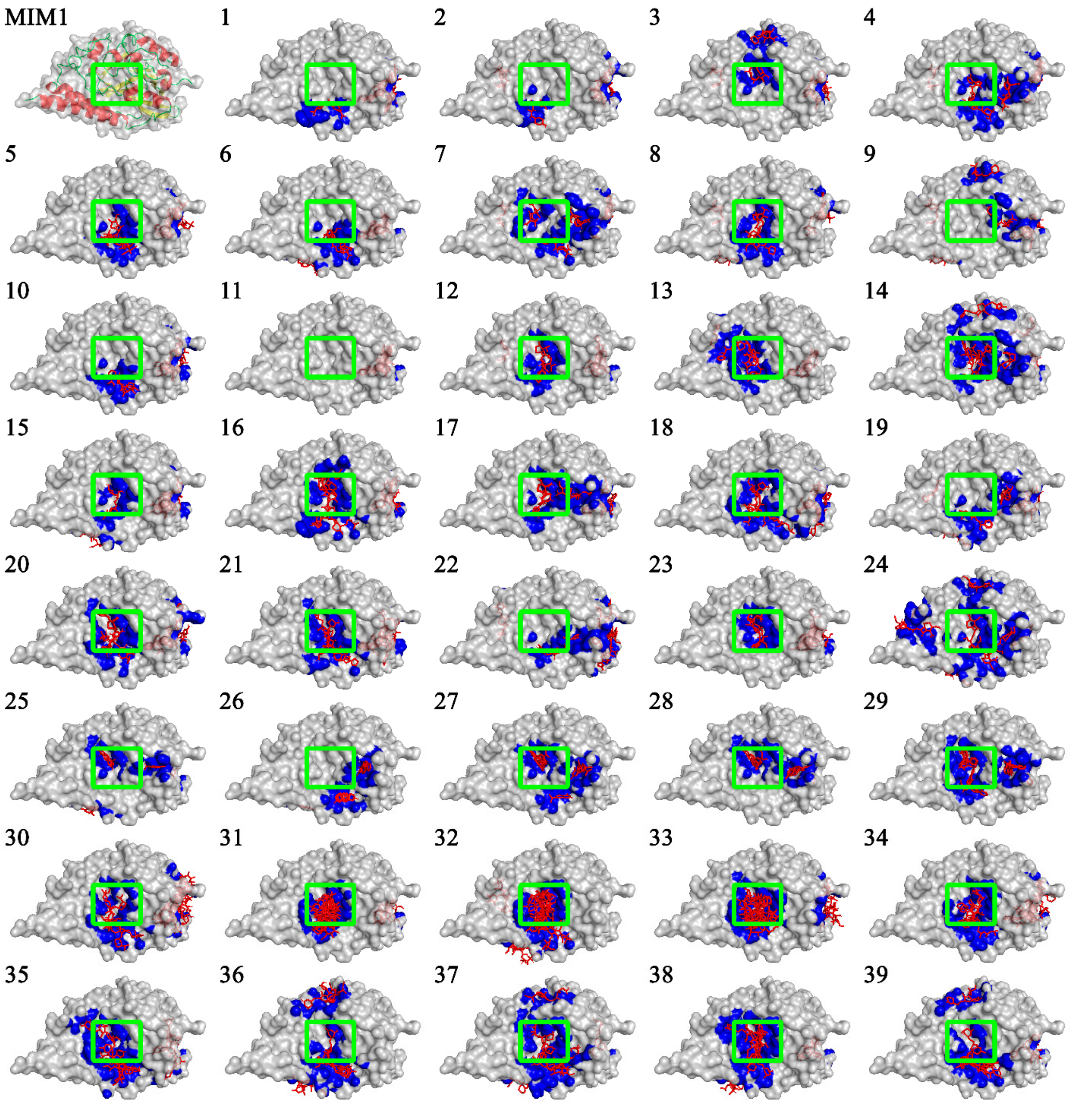

Figure S6. Localization of different ligand molecules (numbering 1–39 is given in the caption to Figure S1) on the surface of enzyme **MIM1**, obtained by computational studies of molecular interactions using molecular docking: the enzyme surface is shown in gray; ligand molecules are highlighted in red; blue color indicates the positions of ligand molecules within a distance of less than 4 Å from the enzyme surface. The catalytic center of the enzyme is outlined by a green rectangle.

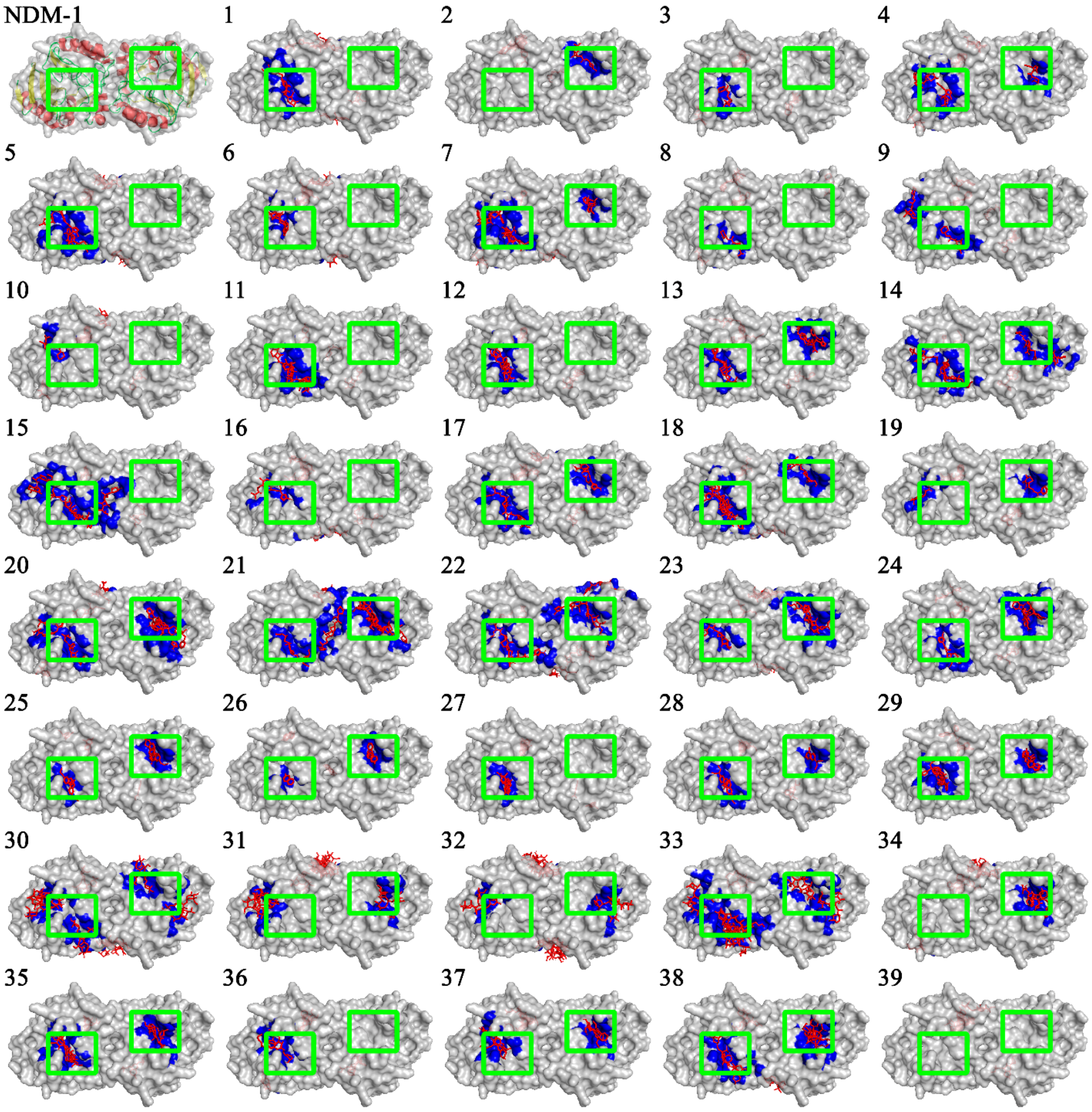

Figure S7. Localization of different ligand molecules (numbering 1–39 is given in the caption to Figure S1) on the surface of enzyme **NDM-1**, obtained by computational studies of molecular interactions using molecular docking: the enzyme surface is shown in gray; ligand molecules are highlighted in red; blue color indicates the positions of ligand molecules within a distance of less than 4 Å from the enzyme surface. The catalytic center of the enzyme is outlined by a green rectangle.

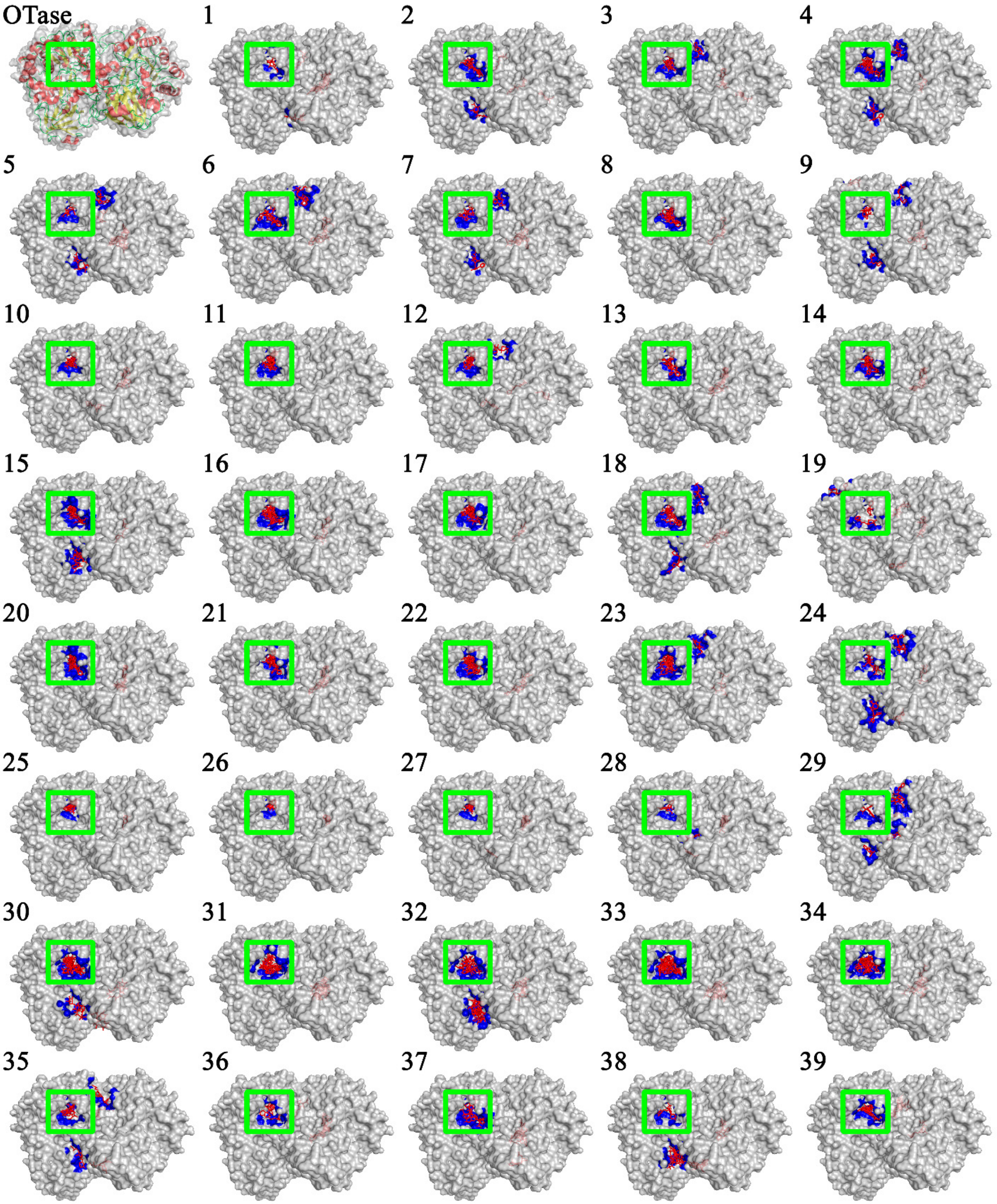

Figure S8. Localization of different ligand molecules (numbering 1–39 is given in the caption to Figure S1) on the surface of enzyme **OTase**, obtained by computational studies of molecular interactions using molecular docking: the enzyme surface is shown in gray; ligand molecules are highlighted in red; blue color indicates the positions of ligand molecules within a distance of less than 4 Å from the enzyme surface. The catalytic center of the enzyme is outlined by a green rectangle.

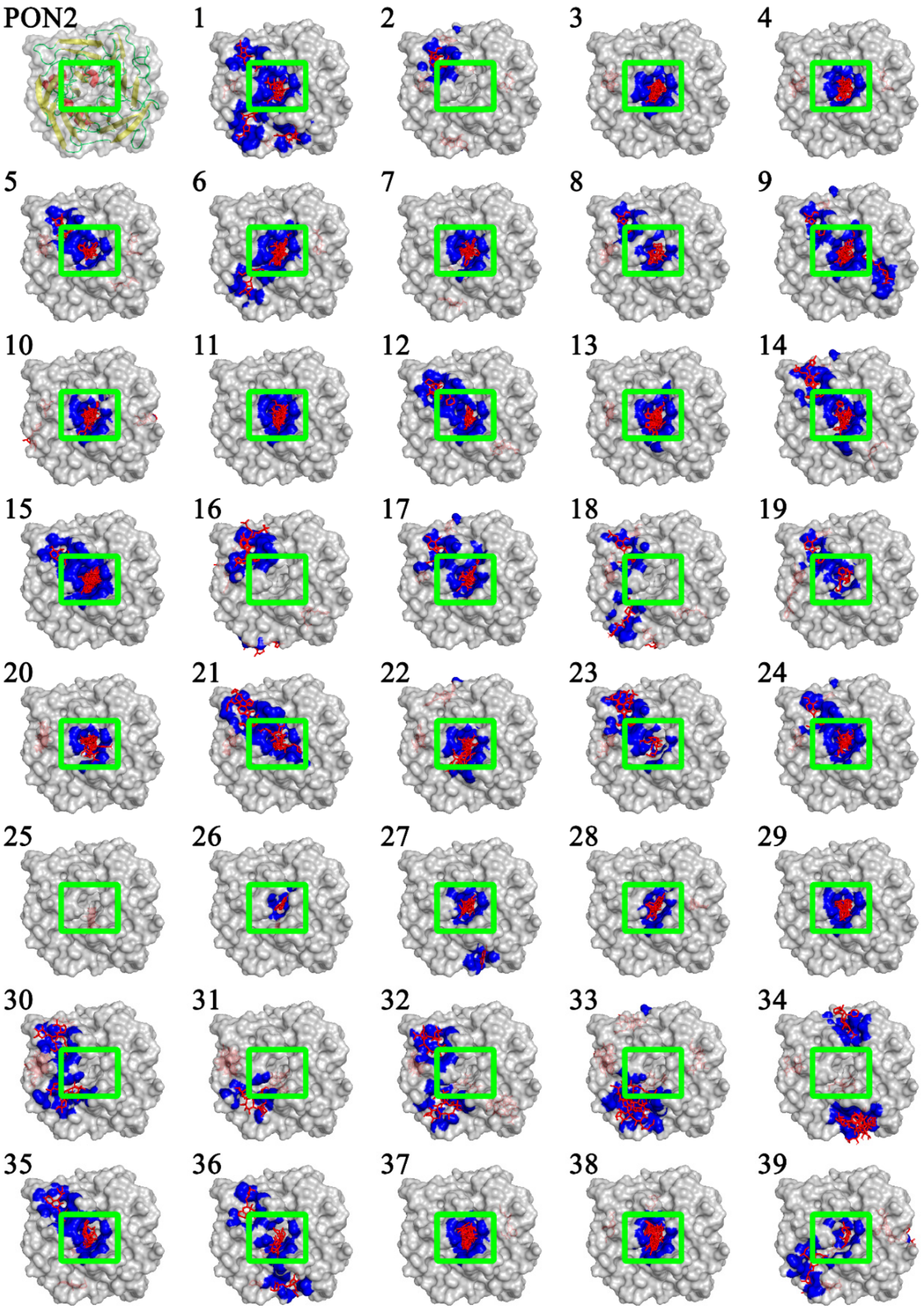

Figure S9. Localization of different ligand molecules (numbering 1–39 is given in the caption to Figure S1) on the surface of enzyme **PON2**, obtained by computational studies of molecular interactions using molecular docking: the enzyme surface is shown in gray; ligand molecules are highlighted in red; blue color indicates the positions of ligand molecules within a distance of less than 4 Å from the enzyme surface. The catalytic center of the enzyme is outlined by a green rectangle.

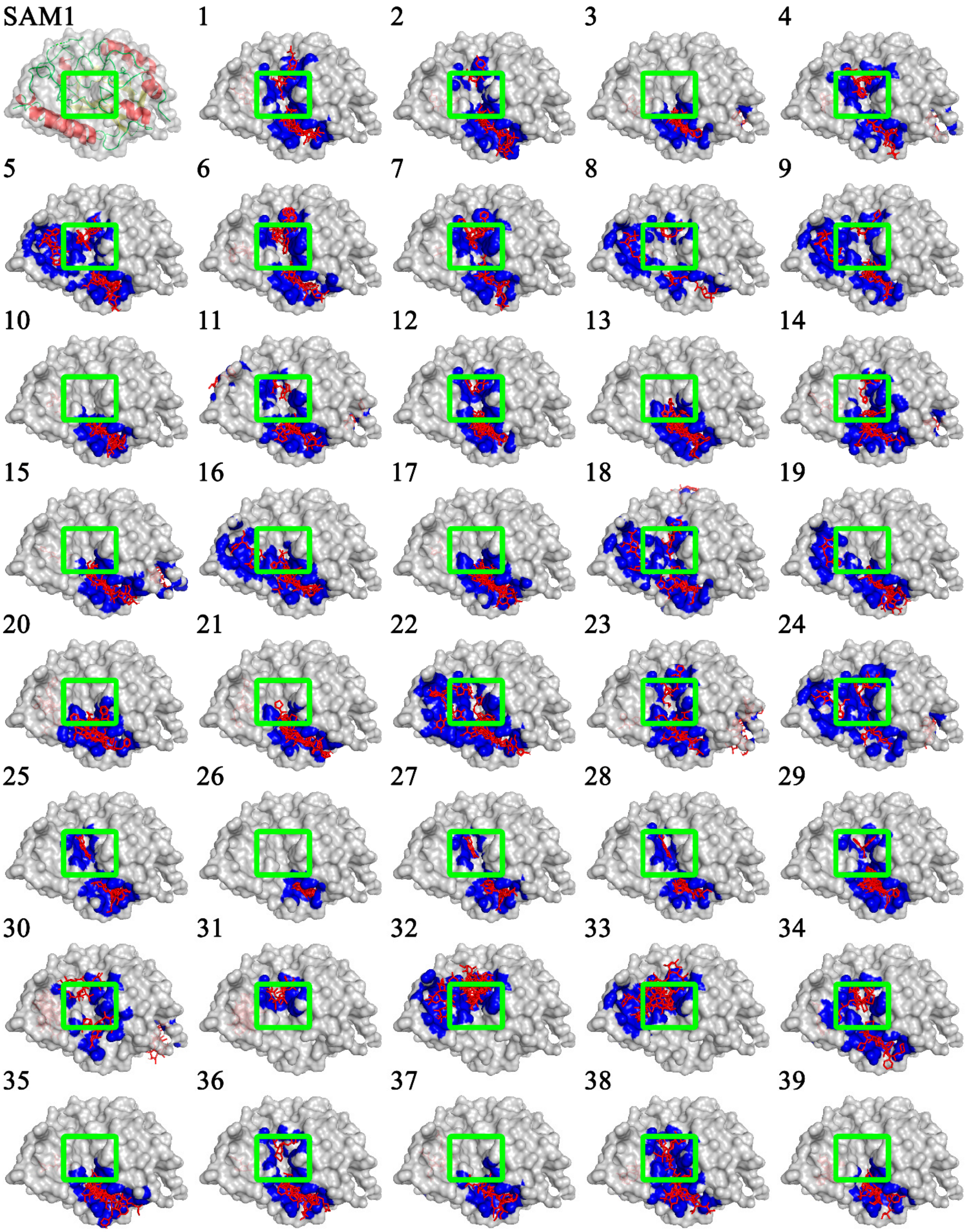

Figure S10. Localization of different ligand molecules (numbering 1–39 is given in the caption to Figure S1) on the surface of enzyme **SAM1**, obtained by computational studies of molecular interactions using molecular docking: the enzyme surface is shown in gray; ligand molecules are highlighted in red; blue color indicates the positions of ligand molecules within a distance of less than 4 Å from the enzyme surface. The catalytic center of the enzyme is outlined by a green rectangle.

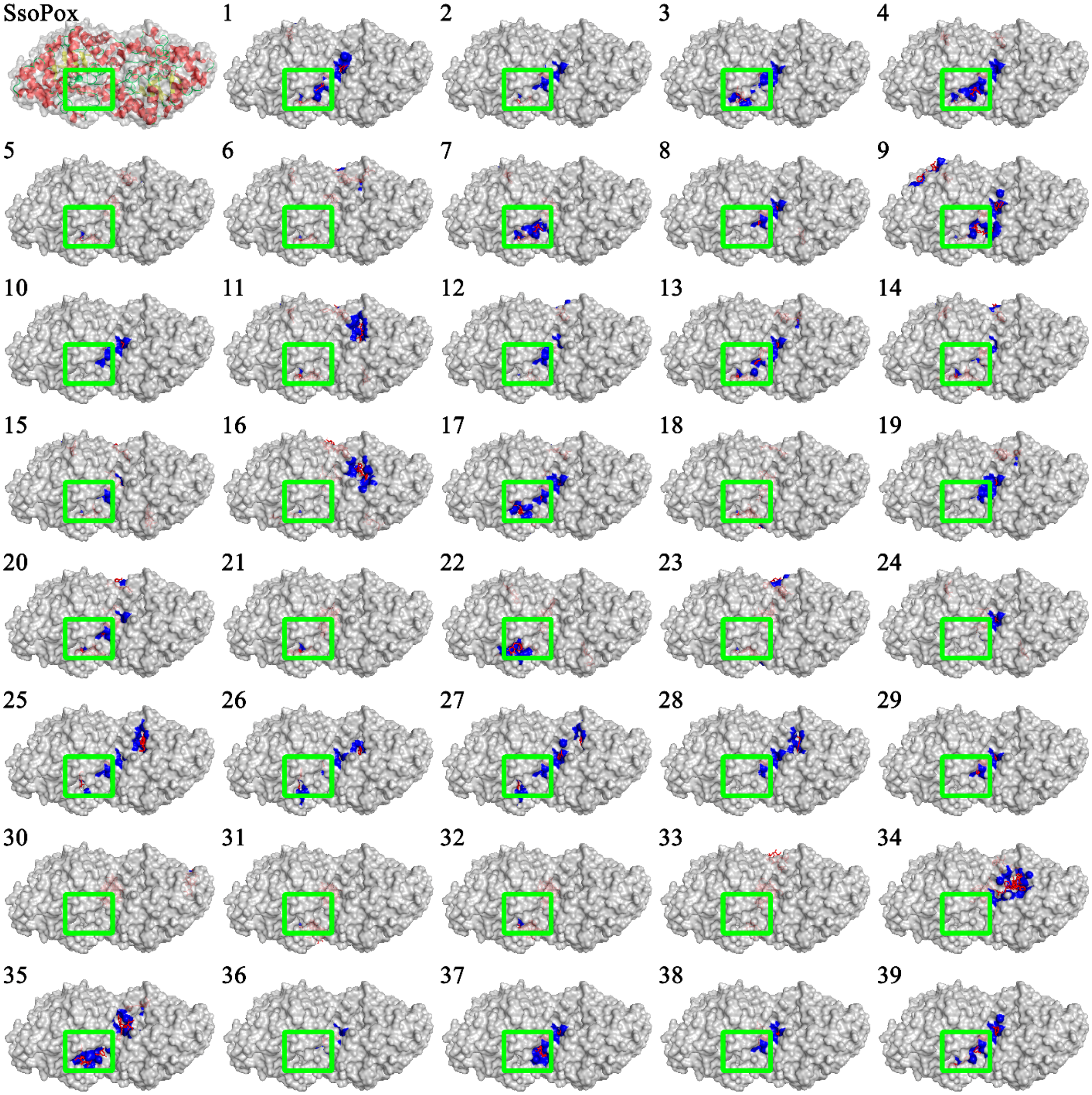

Figure S11. Localization of different ligand molecules (numbering 1–39 is given in the caption to Figure S1) on the surface of enzyme **SsoPox**, obtained by computational studies of molecular interactions using molecular docking: the enzyme surface is shown in gray; ligand molecules are highlighted in red; blue color indicates the positions of ligand molecules within a distance of less than 4 Å from the enzyme surface. The catalytic center of the enzyme is outlined by a green rectangle.

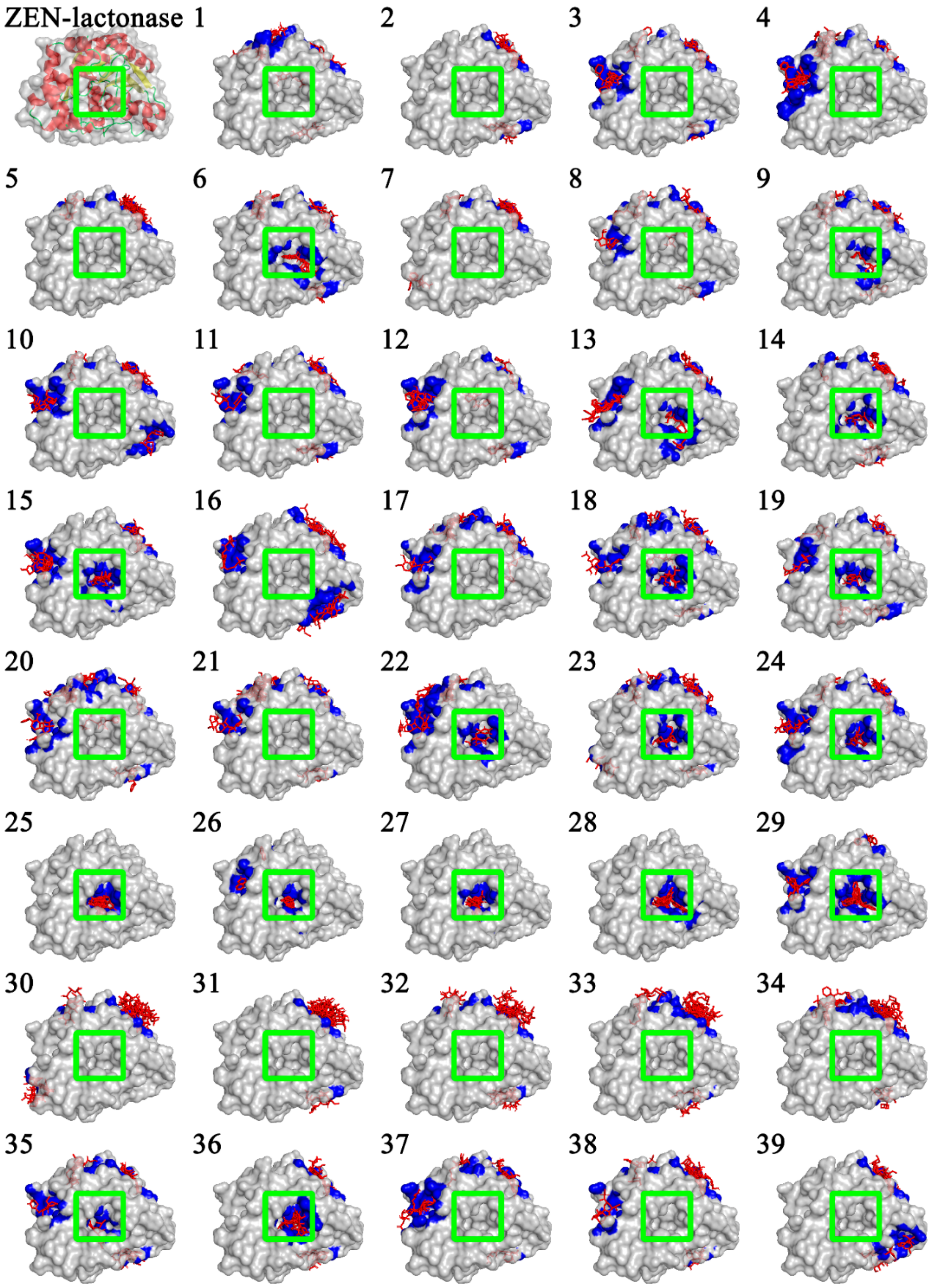

Figure S12. Localization of different ligand molecules (numbering 1–39 is given in the caption to Figure S1) on the surface of enzyme **ZEN-lactonase**, obtained by computational studies of molecular interactions using molecular docking: the enzyme surface is shown in gray; ligand molecules are highlighted in red; blue color indicates the positions of ligand molecules within a distance of less than 4 Å from the enzyme surface. The catalytic center of the enzyme is outlined by a green rectangle.

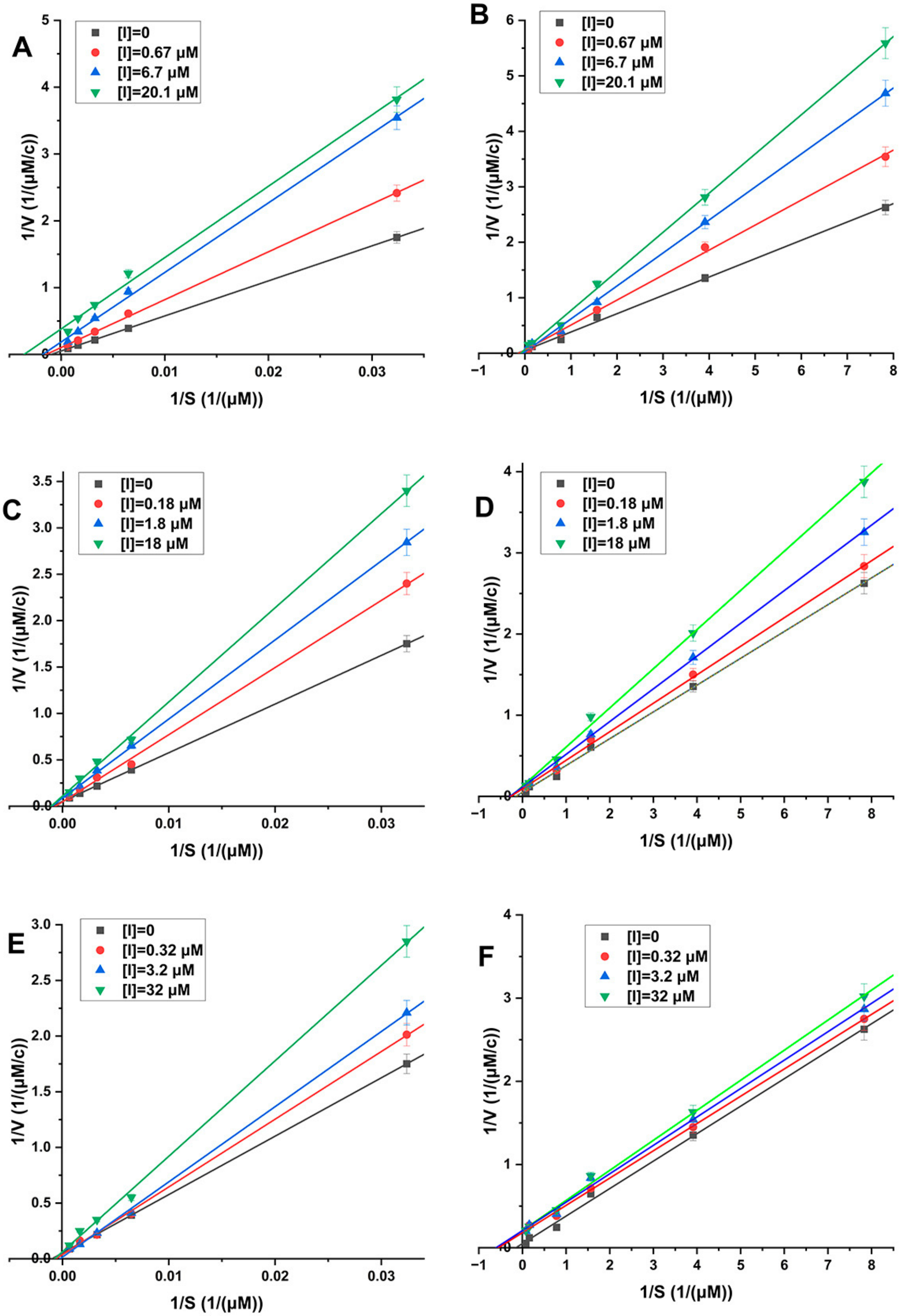

Figure S13. Dependence of the initial rate of the enzymatic reaction on substrate concentration in Lineweaver–Burk coordinates for NDM-1 (A, C, E) and His_6-OPH (B, D, F) in the absence and presence of the inhibitors azithromycin (A, B), simvastatin (C, D), and warfarin (E, F).
